# The Role of Bile Acids in the Human Body and in the Development of Diseases

**DOI:** 10.3390/molecules27113401

**Published:** 2022-05-25

**Authors:** Yulia Shulpekova, Maria Zharkova, Pyotr Tkachenko, Igor Tikhonov, Alexander Stepanov, Alexandra Synitsyna, Alexander Izotov, Tatyana Butkova, Nadezhda Shulpekova, Natalia Lapina, Vladimir Nechaev, Svetlana Kardasheva, Alexey Okhlobystin, Vladimir Ivashkin

**Affiliations:** 1 Sechenov First Moscow State Medical University (Sechenov University), 119435 Moscow, Russia; shulpekova_yu_o@staff.sechenov.ru (Y.S.); zharkova_m_s@staff.sechenov.ru (M.Z.); tkachenko_p_e@staff.sechenov.ru (P.T.); tikhonov_i_n@staff.sechenov.ru (I.T.); lapina_n_n@student.sechenov.ru (N.L.); nechaev_v_m@staff.sechenov.ru (V.N.); kardasheva_s_s_@staff.sechenov.ru (S.K.); okhlobystin_a_v@staff.sechenov.ru (A.O.); ivashkin_v_t@staff.sechenov.ru (V.I.); 2 Biobanking Group, Branch of Institute of Biomedical Chemistry “Scientific and Education Center”, 119435 Moscow, Russia; aleks.a.stepanov@gmail.com (A.S.); izotov.alexander.ibmc@gmail.com (A.I.); t.butkova@gmail.com (T.B.); 3 National Medical Research Center of Endocrinology, 117292 Moscow, Russia; nadshul@gmail.com

**Keywords:** bile acids, bile acids receptors, pathogenesis

## Abstract

Bile acids are specific and quantitatively important organic components of bile, which are synthesized by hepatocytes from cholesterol and are involved in the osmotic process that ensures the outflow of bile. Bile acids include many varieties of amphipathic acid steroids. These are molecules that play a major role in the digestion of fats and the intestinal absorption of hydrophobic compounds and are also involved in the regulation of many functions of the liver, cholangiocytes, and extrahepatic tissues, acting essentially as hormones. The biological effects are realized through variable membrane or nuclear receptors. Hepatic synthesis, intestinal modifications, intestinal peristalsis and permeability, and receptor activity can affect the quantitative and qualitative bile acids composition significantly leading to extrahepatic pathologies. The complexity of bile acids receptors and the effects of cross-activations makes interpretation of the results of the studies rather difficult. In spite, this is a very perspective direction for pharmacology.

## 1. Introduction

Bile acids (BAs) are cholesterol metabolites that are produced exclusively in the liver in a complex multistep process involving cytosolic, mitochondrial, and peroxisomal enzymes [1]. The widespread occurrence of BAs and their derivatives throughout the animal kingdom emphasizes the wide variety of biological functions that they perform in the body. In addition to their “classical” functions (formation of bile, absorption of food lipids in the intestine, proteolytic degradation of food proteins, antimicrobial activity, maintenance of homeostasis), it has been established that bile acids perform hormone-like functions in the control of glucose, lipid, and energy metabolism, in cell proliferation, in the control of detoxification reactions, as well as in the modulation of the immune system [2].

The identification of BAs receptors in the tissue of the cardiovascular system and the recognition of BAs as vasoactive ligands that regulate vascular tone and myocardial contractility in disease have become the reason for the relevance of studying the role of BAs in the regulation of cardiovascular function [3]. It is assumed that in liver diseases (for example, cirrhosis), several mechanisms and ligands, including BAs, induce systemic and internal vasodilation, which causes hyperdynamic circulation. In addition, farnesoid X receptor (FXR) activation may play a role in the development of atherosclerosis. Therefore, the study of the effect of BAs on the cardiovascular system is likely to provide new mechanistic ideas about their regulatory role.

In addition, BAs also play a certain physiological role in the central nervous system [4]. BAs are readily bioavailable when administered orally, subcutaneously, or intravenously, can cross the blood–brain barrier, are relatively non-toxic, and have been approved by the US Food and Drug Administration for therapeutic use in humans [5].

The composition of the BAs pool is a function of the microbial metabolism of bile acids in the intestine. Disruptions to the microbiota form a pool of BAs and modulate the activity of receptors activated by bile acid, even outside the gastrointestinal tract, triggering various metabolic axes and altering the metabolism of the host. BAs, in turn, can also regulate the composition of the gut microbiome at the highest taxonomic levels [6].

The gastric mucosa, colon, and hepatocytes are affected by BAs [7]. However, their toxicity to the mucous membrane of the respiratory tract is not well understood [8]. Patients with a range of respiratory diseases have been shown to have higher BAs concentrations in their bronchoalveolar lavage [9]. In the study [7], it was shown that BAs present in the lungs can cause damage to the epithelium of the airways, which may be a mechanism of lung damage.

In this review, the role of BAs in the regulation of inflammatory reactions, cardiovascular and nervous systems, pathogenesis of liver diseases and the effect on the development of the tumor process, the interaction of BAs with intestinal microbiota, and aspects of the participation of BAs in the pathogenesis of intestinal and pulmonary diseases are considered in detail.

An analysis of the literature was carried out using the PubMed, Mendeley, and Scopus databases. The depth of the literary search was 10 years using additional sources.

## 2. The Role of Bile Acids in the Regulation of Inflammatory Reactions

BA receptors, such as FXR, G protein-coupled bile acid receptor 1 (GPBAR1), vitamin D receptor (VDR), and liver X receptor (LXR), are widely represented in immune cells, monocytes and macrophages, dendritic cells, natural killer cells, and natural killer T cells, and to a lesser extent in T and B lymphocytes. By interacting with these receptors, BAs can influence the course of inflammatory reactions. FXR/small heterodimer partner (SHP) activation blocks the c-Jun N-terminal kinase (JNK) cascade and prevents the binding of nuclear factor kappa B (NF-κB) to the promoters of genes encoding proinflammatory cytokines, chemokines, and inducible NO synthase.

FXR stimulates the binding of the nuclear receptor co-repressor 1 to the promoter of proinflammatory genes, which makes it difficult for NF-κB to interact with them [10]. Under conditions of FXR activation and stimulation of toll-like receptor-4/9 by the corresponding ligands, that is, lipopolysaccharides and CpG regions of bacterial and viral DNA, the inflammatory response of intestinal macrophages is suppressed [11,12]. Probably, the anti-inflammatory effect of more amphiphilic BAs, characterized by a higher affinity for FXR, contributes to the maintenance of the tolerogenic phenotype of intestinal and hepatic macrophages and makes a significant contribution to the suppression of the inflammatory response [12].

In contrast, GPBAR1 activation entails JNK activation with an increase in the expression of genes encoding proinflammatory cytokines interleukin-1β (IL-1β) and tumor necrosis factor-α (TNF-α) [13]. Stimulation of GPBAR1 leads to an increase in the cAMP content in the cell and activation of protein kinase A, mTOR, and nuclear factor FOXO1, which leads to a decrease in the expression of various chemokines. Another cascade involves phosphorylation of the cAMP response element-binding protein and increased expression of the anti-inflammatory cytokine interleukin-10 (IL-10) [12].

Intestine and liver macrophages express GPBAR1 in large amounts, suggesting the important role of non-polar BAs (deoxycholic acid, DCA; taurolithocholic acid, tauro-LCA) in suppressing the inflammatory process in the colon and liver, especially in conditions of increased concentration of non-polar BAs [13,14]. The phenotype of intestinal and hepatic macrophages (M1 or M2) may depend largely on the BA spectrum in the intestinal lumen and portal blood [14].

GPBAR1 is also widely present in the endothelium, especially in the sinusoid endothelium [14]. Here, the role of non-polar BAs in the regulation of NO production and the intensity of hepatic blood flow is of particular importance [15].

In the endothelium, GPBAR1 also inhibits the binding of NF-κB to the promoter of target genes, causing inhibition of the production of vascular-cellular adhesion molecule-1 and TNF-α, inhibiting adhesion of circulating monocytes to endothelial cells [15].

A low degree of GPBAR1 expression is associated with a more pronounced reaction of damage and inflammation in the liver. In GPBAR1 knockout mice, alcohol-induced liver damage is more pronounced, and the negative influence of intestinal dysbiosis is more pronounced [12]. In the GPBAR1 knockout mouse model, the development of prolonged cholestasis, an increased inflammatory response, and severe liver damage was noted in response to partial hepatectomy [16]. In the mouse model of xenobiotic-induced sclerosing cholangitis, the liver injury response was less severe when GPBAR1 was highly expressed [17]. Studies have also shown that stimulation of GPBAR1 helps to reduce the degree of damage to the intestinal mucosa and stimulates regeneration [18].

The VDR signaling pathway plays an important role in the regulation of inflammatory responses, cell proliferation, and apoptosis, as well as in the control of glycolipid and energy metabolism [19]. Animal studies have shown that VDRs are involved in gut immune regulation and barrier function and reduce inflammatory responses [20]. Moreover, some studies have demonstrated the important role of BAs signaling through the VDR in adaptive immunity [21,22], especially in relation to inflammatory bowel disease. The pathophysiological significance of VDR was demonstrated in an experiment where mice lacking VDR were more vulnerable to dextran sulfate-induced colitis [2].

Liver X receptors (LXRα and LXRβ) are members of the family of nuclear transcription factor receptors that play an important role in the transcriptional control of lipid metabolism.

The significance of LXR in the biology of macrophages is relevant in the context of atherosclerosis, which is currently recognized as a chronic inflammatory disease, as well as a disorder of lipid metabolism [23]. The accumulation of large amounts of cholesterol in conditions of hypercholesterolemia is a critical step in the transformation of macrophages into foam cells in the early stages of atherogenesis [24]. LXRs are able to lower cellular cholesterol levels by promoting cholesterol efflux through the activation of the ATP-binding cassette family of transporters, which leads to an increase in reverse cholesterol transport. In addition, macrophage LXRs contribute to the reverse cholesterol transport pathway through a mechanism that includes the induction of a subset of apolipoproteins capable of serving as acceptors for cholesterol remodeling enzymes and lipoproteins [25].

The study of the course of the inflammatory processes in conditions of not only increased but also decreased content of non-polar BA is of great importance.

## 3. The Effect of Bile Acids on the Cardiovascular System

GPBAR1 is widely present in the vascular endothelium and smooth myocytes. A feature of the effects of GPBAR1 stimulation is a systemic vasodilating effect due to an increase in the activity of NO synthase and cystathionine γ-lyase, producing NO and H_2_S, in the endothelium of arterioles and venules [12]. The vasodilating effect is associated with more pronounced lipophilicity of BAs. The vasodilating effect of BAs, independent of GPBAR1 stimulation and NO release by the endothelium, has also been described. Lipophilic BAs likely contribute to a decrease in the affinity of α1-adrenoreceptors for catecholamines due to the activation of lipid peroxidation and a decrease in the plasticity of smooth muscle cell membranes [26].

GPBAR1, expressed by macrophages, appears to play an important role in suppressing inflammation in atherosclerotic plaques and visceral adipose tissue, as well as in promoting lipid transport in adipose tissue [27,28].

The effects of the stimulation of FXR expressed by vascular smooth muscle cells by primary BAs are described; they lead to an improvement in the serum lipid profile and a decrease in vascular tone [29].

**Figure 1:**
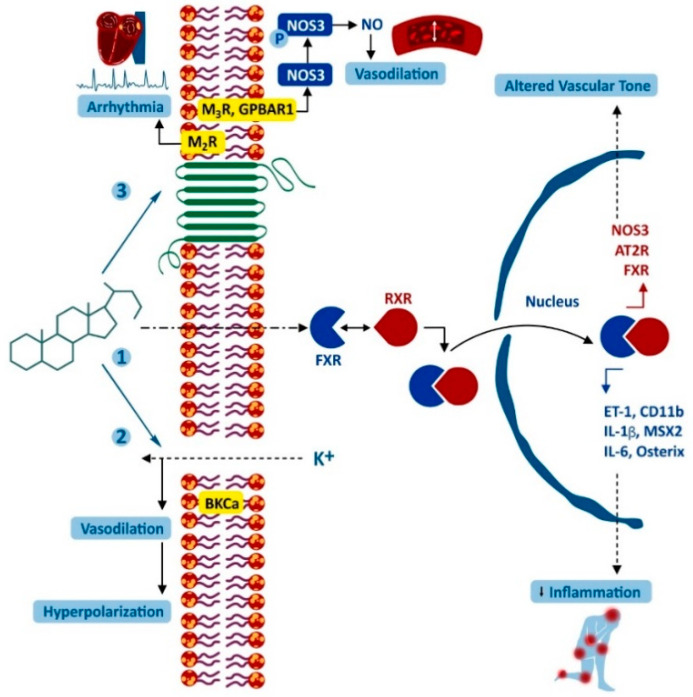
Interaction of bile acids with various cellular molecules: non-receptor-mediated interaction of bile acid with big potassium, calcium-activated channels, which leads to K+ outflow, hyperpolarization, and relaxation of vascular smooth muscles (1) and interaction of bile acid with nuclear receptors (2). In the cytoplasm, bile acid binding to FXR triggers dimerization with RXR, which leads to translocation of FXR into the nucleus, where FXR binds to regulatory elements of the target gene. Downward (red) and upward (blue) arrows indicate down and up-regulation of molecules, respectively. MSX2 and osterix are osteogenic transcription factors. Interaction of bile acid with GPCR, which can lead to a negative chronotropic response (for M_2_R) in cardiac myocytes and the generation of NO (for M_3_R and GPBAR1) in endothelial cells (3). AT2R: type 2 angiotensin receptor; BKCa: large channels activated by potassium and calcium; CD11b: cluster of differentiation 11b; ET-1: endothelin-1; interleukin-1 and -6; M_2_R: muscarinic receptor subtype 2; M_3_R: muscarinic receptor subtype 3; MSX2: homeobox muscle segment 2; NOS3: nitric oxide synthase 3. Figure adapted with permission from Ref. [3] 2011, Khurana, S. et al.

Dysfunction of the heart in liver diseases is associated with increased BAs in the blood. The features of such damage have been studied by ligating the common bile duct in rodents [30]. Cardiomyocyte mitochondria perform numerous functions, including energy production, cell growth, calcium transport, regulation of cell death, and production of reactive oxygen species. Many of these functions are impaired by exposure to toxins and diseases. Consequences of mitochondrial dysfunction in the heart include myocardial infarction and ischemia/reperfusion injury, cardiomyopathies, and arrhythmias, and mitochondrial abnormalities have also been described in the pathogenesis of heart failure [31]. Mitochondrial dysfunction and apoptosis of cardiomyocytes have been observed; these mechanisms likely contribute to the formation of the so-called cirrhotic cardiomyopathy [30,32]. Under conditions of obstructive jaundice, the development of bradycardia caused by the negative chronotropic effect of cholic acid (CA), is characteristic [33]. Several studies suggest that more lipophilic BAs (LCA, DCA, and chenodeoxycholic acid, CDCA) can disrupt mitochondrial function by reducing membrane potential, inducing the formation of mitochondrial permeability transition pores, and the deployment of the programmed cell death cascade [30,34,35]. The more hydrophilic glyco- and tauro-conjugates of these BAs exhibited less damaging effects. Ursodeoxycholic acid (UDCA) and its conjugates had the lowest mitochondrial toxicity index [30]. Figure 1 schematically shows the interaction of BAs with various cellular molecules.

Elevated serum bile acids reduce cardiac fatty acid oxidation both in vivo and ex vivo probably due to suppressed expression of proliferator-activated receptor-γ coactivator 1α (PGC1α), a transcriptional activator of mitochondrial biogenesis, respiratory capacity, oxidative phosphorylation, and fatty acid β-oxidation [36]. In isolated rat heart mitochondria, the most lipophilic BAs (LCA, DCA, and CDCA) showed the most potent inhibition of respiration, respiratory control ratio, and membrane potential and caused the induction of the mitochondrial permeability, while the glycochenodeoxycholic acid possessed the lowest toxicity [37]. These results indicate that at toxicologically relevant concentrations, most bile acids (mainly the most lipophilic) alter mitochondrial bioenergetics. The impairment of cardiac mitochondrial function may be an important cause of the observed cardiac alterations during cholestasis.

Studies on atrial myocardial preparations revealed that an increased concentration of CA can increase the excitability of cells and induce rhythm disturbances. In isolated cardiac myocytes, tauro-CA caused spontaneous depolarization and increased the intensity of the Na^+^/Ca^2+^ flow [38].

## 4. The Influence of Bile Acids on the Hypothalamic–Pituitary–Adrenal Axis

Steroid-producing cells are particularly susceptible to the effects of BAs. The adrenal cortex cells express at least 3 types of BA receptors, namely, FXR, GPBAR1, and sphingosine-1-phosphate receptor 2 (S1PR2). In an experiment, under the conditions of ligation of the common bile duct, oral administration of CDCA significantly increased the activity of steroidogenesis enzymes and the content of corticosterone. Tauro-CDCA has a stimulating effect on the S1PR2-ERK-SF-1 signaling pathway in adrenal cortex cells and on cortisol secretion [39]. At the same time, in cholestatic diseases, there are signs of suppression of the hypothalamic–pituitary–adrenal axis, which is probably associated with increased BAs in the blood. Decreased adrenal function is likely to have an aggravating effect on adaptive responses in these patients. Studies on the subcellular fraction and hepatoma cell culture have shown that the effect of CDCA (simulating cholestasis conditions) reduces the activity of 5β-reductase at the transcriptional and post-transcriptional levels, altering the clearance of steroid hormones. The activity of progesterone, testosterone, cortisol, and aldosterone depends on the activity of this enzyme. BAs suppress the enzyme 11β-hydroxysteroid dehydrogenase, which converts cortisol into cortisone, which probably contributes to sodium retention [40]. Patients with cholestasis show decreased urinary excretion of 3α, 5β-tetrahydrocortisol, and decreased adrenal mass. A low-fat diet helps reduce the degree of cholemia and increases the activity of 5β-reductase in the liver; however, in real practice, this measure is not applicable [41]. The function of cortisol receptors on hepatocytes is of great importance in the regulation of BAs metabolism. Experiments in mice with a defect in the cortisol receptor showed a decrease in sodium taurocholate cotransporting polypeptide (NTCP) activity and BA import into the hepatocyte, reduced content of BAs in bile, frequent formation of gallstones, the development of steatorrhea, and weight loss. Cholemia that develops as a result of NTCP inhibition stimulates heat production in brown adipose tissues. Similar changes in the metabolism of BAs and clinical manifestations can be observed in chronic insufficiency of the adrenal cortex (Addison’s disease) due to insufficient production of glucocorticoids [42].

## 5. The Role of Bile Acids in the Nervous System

Cells of the central nervous system express receptors that interact with which BAs and include nuclear receptors FXR, VDR, glucocorticoid receptor (GR), membrane receptors GPBAR1, M2/3, and S1PR2 [43,44].

**Figure 2:**
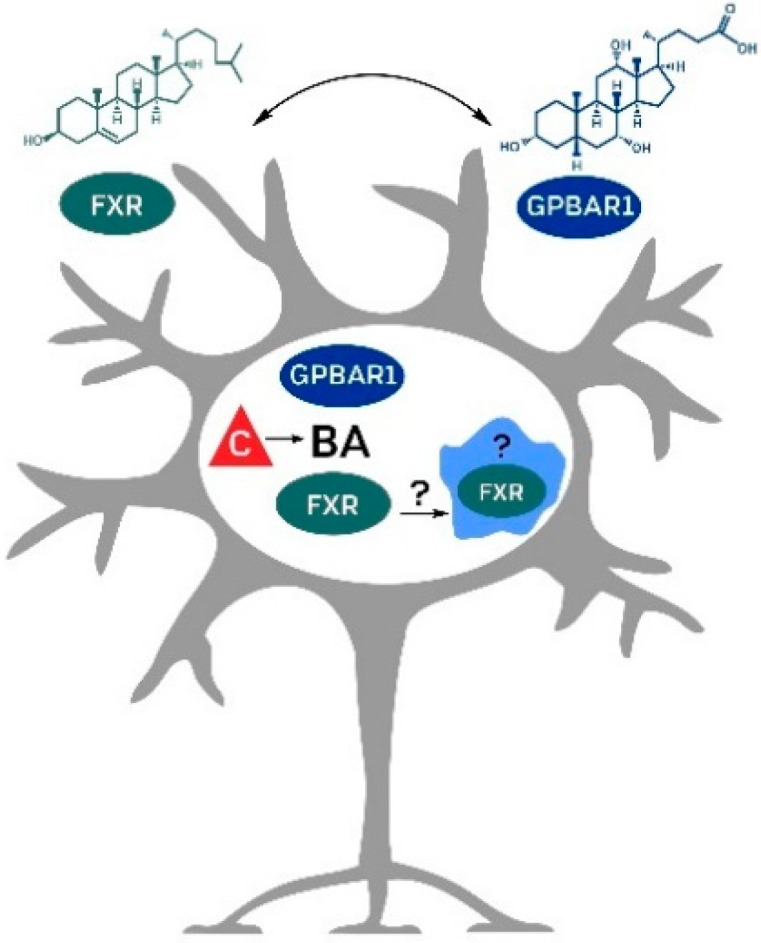
Possible primary signaling pathways (GPBAR1 and FXR) and the potential role of endogenous bile acids in models of neurodegenerative disorders. BA—bile acids, C—cholesterol, FXR—farnesoid X receptor, GPBAR1—G protein-coupled bile acid receptor 1.

In the central nervous system, the function of FXR is not well understood (Figure 2).

An FXR-knockout mouse model with a shutdown of the mechanism for suppressing the synthesis of BAs in the liver increased BAs content in the blood and a significant increase in the content of secondary BAs and their conjugates with taurine in the central nervous system. It also led to reduced severity of anxiety reactions, decreased memory, increased motor activity, and impaired coordination of movements. Changes in glutamatergic, γ-aminobutyric acid (GABA)-ergic, serotoninergic, and noradrenalinergic transmission in the hippocampus and cerebellum were also recorded. In the hippocampus, a decreased content of the enzyme involved in the formation of GABA was noted, while in the cortex, the activity of the transporter of this mediator increased. Therefore, FXR in the nervous system is involved in the regulation of neurotransmitter activity [44].

VDRs are localized in neurons and glia of the areas of the brain involved in complex planning, information processing, and amnestic activity (in particular, in the temporal lobe, cingulate gyrus, visual cortex, thalamus, amygdala), as well as in Purkinje cells [45]. VDR stimulation induces the expression of calcium-binding proteins parvalbumin and calbindin, inhibits the function of calcium channels in the hippocampus, and prevents excitotoxic reactions [46]. Another important effect of VDR stimulation is the production of anti-inflammatory cytokines IL-4 and transforming growth factor TGFβ in neuroglia [47]. VDR, as well as FXR, can be activated by secondary bile acid LCA [48]. Considering the importance of these effects, it is worthwhile to study the effects of the interaction of VDR with BA in the nervous system. The structures of LCA and 1α,25-dihydroxyvitamin D3 differ significantly; however, both ligands bind to the same ligand-binding pocket of VDR, but in the opposite orientation [49]. VDR plays an important role in autophagy [50]. Calcitriol activates autophagy by inducing *ATG16L1* expression, which also provides anti-inflammatory effects and improved lipid profiles [51]. Other important aspects of VDR activation by calcitriol are mitophagy [52] and intestinal antimicrobial defense [53]. VDR interacts with hepatocyte nuclear factor 4-alpha leading to the inhibition of CYP7A1 gene transcription and decreased bile acid synthesis. This aspect is important for liver cell protection in cholestasis [54]. As all these facts were established for calcitriol, the studying of VDR-mediated effects of LCA seems to be of great importance.

The chemical structure of BAs is very similar to that of neurosteroids. Neurosteroids are predominantly produced in neurons and glial cells of the cerebral cortex, subcortical white matter, and hippocampus. The type A GABA (GABA_A_) receptor, the main channel of inhibitory impulses in the central nervous system, is the main target of neurosteroids. Neurosteroids also modulate the functions of N-methyl-D-aspartic acid (NMDA), the α-amino-3-hydroxy-5-methyl-4-isoxazolepropionate acid receptor, as well as the kainate, σ-receptor, glycine, serotonin, nicotinic and muscarinic receptors. Neurosteroids have a pronounced anticonvulsant, sedative, antinociceptive, and antidepressant effect, and affect the mechanisms of learning and memory [55]. These substances also interact with GPBAR1 expressed on astrocytes and neurons; as a result of adenylate cyclase activation, an increase in the intracellular Ca^2+^ content and the production of reactive oxygen species are observed [56]. BAs are also able to modulate the function of GABA_A_ and NMDA receptors, acting as their antagonists. The maximum antagonism with respect to GABA_A_ and NMDA receptors was observed in CDCA, followed by DCA, and CA. UDCA acts on GABA_A_ receptors while decreasing drowsiness by disinhibiting the histaminergic system [57,58]. Glyco- and tauro-derived UDCA acids exhibit a neuroprotective effect by inhibiting excessive stimulation of glutamate receptors and associated excitotoxic reactions in the hippocampal tissue [59]. The same highly hydrophilic conjugated BAs protect the endothelium of the blood–brain barrier from oxidative damage [60], while hydrophobic secondary ones, damage interendothelial contacts [61]. A high total BAs content in the blood has been shown to change the physical properties of endothelial membranes in vitro, which leads to the development of arterial hypotension leading up to refractoriness to the administration of vasopressors [26].

UDCA and its conjugate with taurine through interaction with GPBAR1 have anti-inflammatory effects on astrocytes and microglia, counteracting the activation of NF-κB and decreasing the production of NO and vascular adhesion molecules [62]. In models of neurodegenerative diseases such as Huntington’s disease, Alzheimer’s disease, Parkinson’s disease, acute ischemia, and hemorrhagic stroke, Tuaro-UDCA blocks endoplasmic reticulum stress and prevents neuronal apoptosis [5]. A decrease in the level of BAs or their intermediates in the brain is associated with an increased risk of developing Alzheimer’s disease and Parkinson’s disease [63]. A placebo-controlled phase II study is currently underway to examine the efficacy of UDCA at a dose of 30 mg/kg per day in the treatment of parkinsonism. The theoretical basis for the use of BAs in this study is its potentially beneficial effect on mitochondrial function; it involves performing a functional magnetic resonance imaging of the brain with the isotope of phosphorus 31P to assess the state of ATP metabolism [64].

The issue of the degree of BAs penetration from the bloodstream into the brain, and vice versa, has been insufficiently studied. The permeability of the blood–brain barrier for BAs under physiological conditions has been assessed mainly in animals and suggests that less hydrophilic BAs can penetrate by diffusion, and their content in the brain under physiological conditions correlates with serum BA concentration [65]. Conjugated BAs characterized by a larger molecule and pronounced polarity can be transported by active transport.

In the exchange of BAs, the ependyma of the choroid plexus may play a special role and expresses the apical bile salt transporter (ABST) transporter (mainly in the hypothalamus and the cortex of the frontal lobes) [66,67,68,69]. ABST is present on both the apical and basolateral membranes of the choroid plexus epithelium, which indicates the possibility of bilateral transfer. In addition, on the cells of the endothelium and epithelium of the choroid plexus, other carriers of BAs, namely, multidrug resistance-associated protein 2/4 (MRP2/4), bile salt exporting pump (BSEP), NTCP, have been found and their abundance is approximately 2% of that in the liver [68,70].

The effect of BA on brain cells in liver diseases is of great interest. Experiments involving ligation of the common bile duct in animals, against the background of a decrease in the total BA content in the brain tissue revealed a dramatic increase in the proportion of LCA (≈87%) with toxic potential, which, from the point of view of the authors, explains the origin of encephalopathy in cholestasis [71]. Several researchers suggest that cells that are devoid of BA excretion systems may suffer the most from BA overload [71]. Under these conditions, the most hydrophobic BAs can exhibit a detergent effect on membranes [72]. In cholestatic liver diseases (primary biliary cholangitis, primary sclerosing cholangitis, etc.), BAs are found in the brain tissue, which is traditionally explained by their excessive content in the blood and penetration through the blood–brain barrier [70].

In the model of acute liver failure, a multi-fold increase in the level of BAs in the plasma results in an increase in the content of BAs in the frontal cortex (which may indicate their local synthesis or increased permeability of the blood–brain barrier). Additionally, it also shows an increased expression of NTCP and FXR in the cortex. Artificial blockade of FXR reduces the rate of progression of neurological disorders [66]. In the model of acute hepatic encephalopathy, activation of GPBAR1 is accompanied by a decrease in the secretion of chemokine molecules by neurons and the sensitivity of macrophages of nervous tissue, microglia, to proinflammatory stimuli, which is considered a mechanism for suppressing neuroinflammation [66]. The most important problem is the development of pruritus in cholestatic liver diseases. Because a clear relationship between the concentration of BAs and the intensity of itching has not been established, their pathogenetic significance is questioned. However, therapeutic measures aimed at reducing the degree of cholemia are effective in some patients. Recently, a Mas-related G-protein coupled receptor member X4 has been described, with which BAs can interact and which is expressed in the dorsal ganglia together with the type 1 histamine receptor; activation of Mas-related G-protein coupled receptor member X4 provokes itching [73].

During the cycle of enterohepatic circulation, BAs can influence the brain by activating peripheral receptors FXR and GPBAR1. The FGF19 messenger molecule, formed during the interaction of BA with FXR in the intestine, is transported across the blood–brain barrier and interacts with the FGF4 receptor/β-klotho co-facto [74]. β-Klotho is selectively expressed in the suprachiasmatic, arcuate, and paraventricular nuclei of the hypothalamus, area postrema, and the nucleus of the solitary pathway [74].

The interaction of secondary BAs with GPBAR1, expressed at the endings of peripheral nerves, may contribute to the development of neuropathic pain and the pathogenesis of functional intestinal disorders [75].

## 6. Aspects of the Involvement of Bile Acids in the Pathogenesis of Intestinal Disease

### 6.1. Bile Acids and Intestinal Microbiota

Microbiota of the gastrointestinal tract can be considered a component of the innate immune defense. Stimulation of FXR with bile acids activates the expression of genes for angiogenin (Ang1) and nitric oxide synthase (iNOS), as well as increases the production of antimicrobial peptides cathelicidins by the epithelium through the cascade of extracellular protein kinase (ERK 1/2) [53].

The role of BA in the regulation of the microbial population is significant; reduced BA content is associated with excessive bacterial growth and inflammation [76]. Excessive bacterial growth is accompanied by more intensive deconjugation of primary BA, as a result of which their ability to form micelles decreases and the risk of steatorrhea development increases. In addition, unconjugated BAs are more passively absorbed along the small intestine, bypassing the stage of interaction with FXR expressed in more distal regions; accordingly, the regulatory influence of FXR is significantly reduced [77].

BA can influence the expression of microbial genes encoding virulence factors. In the presence of bile, the expression of the region containing genes of the pathogenicity island of enterohemorrhagic *E. coli* O157:H7 is reduced. As the concentration of bile in the distal small intestine decreases, the bacterium begins to show its virulence again [78]. Adaptive changes in gene expression that contribute to the survival of a microorganism in an aggressive environment have been shown for *Listeria monocytogenes* [79]. The expression of *Salmonella enterica* island of pathogenicity genes required for invasion of the ileal epithelium is also inhibited by BA [80]. Some genes that regulate the degree of virulence of *Shigella*, *Salmonella*, *Vibrio cholerae*, can be activated in the presence of bile salts, which probably serve as a signal of the presence of a favorable intestinal environment for the invasion of these bacteria [81,82]. Changes in gene expression and protein synthesis in the presence of BAs are described in *Campylobacter jejuni* and *Enterococcus faecalis* [83,84]. The regulatory action of BAs on bacteria occurs due to interaction with specific receptors of the bacterial membrane and activation of signaling cascades that regulate gene expression [85]. Tauro-CA interacts with the CspC receptor of *Clostridium difficile* spores and activates their germination, while CDCA has the opposite effect [86,87].

In the colon, less polar BAs (DCA, CDCA, and LCA), to a certain extent likely have an antimicrobial effect due to their ability to diffuse through bacterial membranes [88]. Protonated BAs freely diffuse through the lipid bilayer. Bile acid molecules penetrate the membrane in the form of dimers with a hydrophilic α-part inside and a hydrophobic β-part outside. The bacterial defense mechanism that prevents this process is the maintenance of a higher pH, which leads to the dissociation of protonated BAs resulting in a negative charge [88]. Unconjugated CDCA and DCA, which increase membrane permeability and disrupt the operation of the proton pump have shown a particularly high bactericidal effect against Staphylococcus aureus [89]. The symbiosis of bacteria producing hydrolases and oxidases, as well as the degree of production of short-chain BAs, can affect the spectrum of BAs and the degree of their protonation. The effect of probiotics on the spectrum of BAs and stimulation of the corresponding receptors in the intestine and outside it should be further studied [90].

Microbiota and dietary habits, in turn, significantly affect the total pool and composition of BAs. Mice grown under sterile conditions showed a significant increase in the pool of bile acids due to the synthesis of primary BAs and more active absorption by ABST. This is associated with insufficient stimulation of FXR in the ileum due to the absence of the stage of microbial dehydroxylation of the primary BA β-muricholic acid conjugated with taurine, which is not an FXR agonist [76]. Reduced stimulation of GPBAR1 and decreased production of GLP-1 are associated with an increase in the proportion of primary BAs that have not undergone dehydroxylation, which contributes to an increase in the volume of the gallbladder and the formation of gallstones [91]. People that follow the “Western” style of nutrition show the predominance of secondary BAs in the total pool of BAs, which may explain their increased risk for cancer [92].

Activation of GPBAR1 and FXR contributes to the maintenance of immunological tolerance at the intestinal level and shifts the polarization of macrophages towards the anti-inflammatory M2 phenotype, which produces high concentrations of IL-10. The result of increased production of IL-10 is an increase in the content of FoxP3^+^ in Treg cells and a decrease in the migration of monocytes into the lamina propria of the colon [12]. By acting on GPBAR1 and FXR, BAs indirectly regulate leukocyte migration and differentiation of macrophages in the distal ileum and colon; however, the role of certain types of BAs in this regard is not well understood. In mouse models of colitis, FXR activation has been shown to inhibit the expression of NF-κB-dependent genes (TNF-α, IL-1β, IL-6, COX-1, COX-2, and iNOS genes). The anti-inflammatory effect of FXR manifests through SHP, as well as the stabilization of the nuclear co-repressor NCoR on the NF-κB-sensitive element of the IL-1β gene promoter. Reduced FXR expression is associated with the development of colitis in rodent models as well as in Crohn’s disease [93].

An important aspect of the development of diarrhea in non-inflammatory bowel diseases can be malabsorption of BAs, the signs of which are found in about a quarter to a third of patients who are diagnosed with irritable bowel syndrome with diarrhea or functional diarrhea. The main reason for the development of diarrhea in these cases is decreased ABST function; entry of excessive BAs in the colon stimulates the secretion of chlorides by colonocytes [94]. Confirmatory tests include tests using taurine-75Se-homocholic acid or the assessment of 7α-hydroxy-4-cholesten-3-one in blood serum, reflecting the activity of the classical cascade of BA synthesis [95,96]. As seen within mice, BAs also contribute to the development of visceral hypersensitivity due to stimulation of FXR, the NF-κB cascade, and nerve growth factor (NGF) release from mast cells, as well as increased expression of TRPV1 in the spinal ganglia [97]. Gastrointestinal malabsorption is detected in a significant proportion of patients with microscopic colitis [96]. The treatment plan for such patients includes BA adsorbents [94,96].

The use of FXR ligands seems to be a promising treatment for enterocolitis. 6-ethyl-CDCA, a synthetic ligand of FXR, reduces the degree of macrophage activation by lipopolysaccharide. In vivo application of 6-ethyl-CDCA contributed to a decrease in the degree of activation of immune cells and organ damage [93].

### 6.2. Bile Acids and Colon Tumors

As seen in the study of the effects of DCA and UDCA, depending on the degree of polarity, BAs can differently affect the phases of the cell cycle of the cells of the intestinal epithelium [98]. Ever since the first experiments in mice, secondary BAs, especially DCA, have been considered potential carcinogens [99]. DCA activates the EGFR/MAPK cascade and increases cell survival by activating the signaling molecules AKT, ERK1/2, JNK1,2 COX2, PGE2, and beta-catenin, and may contribute to the progression of colon cancer. The mechanism of action of DCA is not clear; it may manifest through the stimulation of NADP-oxidase, lipid peroxidation due to mitochondrial damage, activation of EGFR due to membrane reorganization, or interaction with FXR [98,100]. The most hydrophobic BAs (particularly, DCA) modify the membrane protein caveolin-1, which is accompanied by dysfunction of various receptors. In particular, ERK1/2 activation is considered a manifestation of caveolar or membrane stress [101].

Subjects at high risk of developing colorectal cancer showed a higher content of DCA and 7α-dehydroxylating bacteria in their feces. High-fat content in food helps to increase the level of DCA in the intestine [102]. Exposure to UDCA and tauro-UDCA is associated with the suppression of colon carcinogenesis. UDCA inhibits the formation of intracellular reactive oxygen species and cell growth, as well as the inflammatory cascade in experimental colitis [103,104].

Thus, DCA and UDCA seem to have different effects on the same oncogenic signaling pathways and must be studied further [98,105]. The findings from several studies are somewhat inconsistent and can be attributed to the peculiarities of the organization of the experiments. In the human colon cancer cell line HCT116, increased DCA and CDCA has been shown to induce apoptosis of cells within a few hours after exposure. Their chemical modifications (in particular, chio-, lago-, nor-DCA), as well as homo-, iso-UDCA, which are less hydrophobic, delay apoptosis. BAs conjugated with glycine or taurine only slow down cell growth [106].

### 6.3. The Gastrointestinal Tract after Cholecystectomy

It is difficult to construct a biological model of BA metabolism after the removal of the gallbladder due to insufficient knowledge of the adaptive mechanisms. Some data indicate that in such patients, BAs accumulate in the proximal small intestine because of the continuous flow of bile, which does not depend on the release of cholecystokinin [107]. A small study assessing the effects of duodenal bile on an empty stomach showed that after cholecystectomy, the BA content in relation to bilirubin increases, and cholesterol decreases by 27%; such changes reflect an increase in the enterohepatic circulation of BA. The synthesis of CA is reduced by 37%, likely as the result of inhibition by the feedback mechanism. The kinetics of BA stabilizes three months after the removal of the gallbladder [108]. The concentration of BAs in the blood is likely determined by intestinal peristalsis and no longer depends on the rhythm of nutrition and contractions of the gallbladder [107]. After cholecystectomy, the serum BA level more than doubles, while there are no pronounced increases in the level of FGF19, which is normally produced in the gallbladder in large quantities [109].

Several studies have shown that cholecystectomy is an independent risk factor for the development and progression of non-alcoholic fatty liver disease (after standardization of data on traditional metabolic risk factors) One of the proposed explanations is the violation of entero-hepatic circulation of BAs, as well as the metabolism of triglycerides due to the increased supply of free BAs from adipose tissue to the liver the influence of BA and is the most important mediator in the reverse regulation of BA synthesis in the liver [109]. Cases of non-alcoholic steatohepatitis after cholecystectomy can also be explained by significant and progressive changes in the composition of the intestinal microbiota (an increase in the population of *Bacteroides ovatus*, *Prevotella copri*, and *Fusobacterium varium*, and decrease in that of *Faecalibacterium prausnitzii* and *Roseburia faecalis*. There exists conflicting data and some studies suggest no effect of cholecystectomy on the progression of non-alcoholic fatty liver disease [109].

A meta-analysis of 10 cohort studies showed that cholecystectomy was associated with an increased risk of colon cancer (hazard ratio 1.30; 95% CI 1.07–1.58) [110]. Another study showed that cholecystectomy was associated with a tendency of the formation of recurrent adenomatous polyps [111,112].

## 7. Possible Effects of Bile Acids on the Growth of Tumor Cells

Patients with colorectal cancer show an increased content of DCA in bile, feces, and serum [92]. The possible involvement of DCA in the development of hepatocellular carcinoma against the background of obesity, tumors of the Vater papilla, and other localizations, have been discussed in several studies; as an important condition for the implementation of the negative effect of DCA is the phenomenon of cellular aging [113,114]. The Western diet high in saturated fat promotes the preferential conjugation of BAs with taurine, which acts as an additional source of organic sulfur for sulfate-reducing pathobiont bacteria, such as *Bilophila wadsworthia*, that play a role in the pathogenesis of colitis and colorectal cancer [115,116].

## 8. The Role of Bile Acids in the Pathogenesis of Liver Diseases

Liver diseases are associated with alterations in the qualitative and quantitative composition of the BA pool. Extrahepatic manifestations, such as muscle hypotrophy, changes in vascular tone, etc., are probably associated with changes in the signaling cascades of BAs. Due to the diversity and variability of the course of liver diseases, many aspects remain insufficiently studied. Tauro-CA, which predominates in the liver under physiological conditions, has shown a protective effect on biliary epithelial cells through activation of the signaling pathway of phosphoinositide 3-kinase (PI3K)/kinase AKT, vascular endothelial growth factor (VEGF), cAMP, and regression damage [117].

Changes in the synthesis of primary BAs and their transport in liver diseases can be explained by oxidative stress and damage to the cytoskeleton with impaired expression of BA transporters on the membrane, changes in blood flow, and other factors [118]. One of the characteristic changes is a change in the activity of enzymes that control the exchange of xenobiotics (constitutive androstane receptor (CAR), pregnane X receptor (PXR)) along the three zones of the hepatic acinus [119]. A significant contribution to the change in the BA spectrum is made by the change in the intestinal microbiota. For example, it has been shown that in liver cirrhosis, the total BA content in feces is reduced, while the proportion of primary BAs is increased, which reflects the inhibition of the conversion of primary BA salts into secondary salts. Such changes develop against the background of an increased content of potentially pathogenic Enterobacteriaceae and a decrease in the population of bacteria producing 7α-dehydroxylase (*Lachonospiraceae, Ruminococcaceae, Blautia*). A significant correlation was shown between the content of these microorganisms and the ratio of primary and secondary BAs (CDCA for *Enterobacteriaceae*, DCA and DCA/CA for *Ruminococcaceae*, and LCA/CDCA for *Blautia*) [120].

A decrease in the total pool of BAs has been described in liver cirrhosis, which probably contributes to the development of bacterial overgrowth in the small intestine and intestinal dysbiosis. The production of proinflammatory cytokines and other mediators of inflammation by the intestinal microbiota, in turn, contribute to the suppression of the synthesis of new BAs. The pathogenesis of complications of liver cirrhosis and portal hypertension are directly related to an increased intake of lipopolysaccharide from the intestine [121].

The study of BA metabolism in cholestatic liver diseases and non-alcoholic fatty liver disease (NAFLD) attracts the most attention. Models for the reproduction of cholestatic liver diseases in animals include those with ligation of the common bile duct, supplemented by vagotomy, ligation of the hepatic artery, and toxic effects (in particular, the effect of carbon tetrachloride). In the model of bile outflow obstruction, damage to cholangiocytes with an increase in their proliferation is reproduced. Vagotomy, the effect of toxins, and ischemia simulate a violation of proliferative activity and the ability of cells to survive an increase in the propensity for apoptosis and a decrease in ductal secretion [117].

Under conditions of cholestasis, protective mechanisms that protect the hepatocyte from overloading with bile acids are triggered—the activity of NTCP and MRP2 decreases, and the activity of MRP3 increases. BSEP function can undergo multidirectional changes, depending on the type of liver damage. For example, in drug-induced cholestasis due to damage to the cytoskeleton, BSEP expression is sharply reduced, usually in conjunction with the expression of the conjugated bilirubin transporter MRP2 [118]. Adaptive changes in cholestasis also include a decrease in the abundance of ABST in the proximal renal tubules and an increase in MRP2 at the apical membrane, which promotes the excretion of BAs in the urine; however, the development of cholemic nephropathy may reduce the relative contribution of this mechanism [122].

The importance of an alternative pathway for BA production significantly increases under conditions of cholestasis due to the activation of FXR/SHP, CAR, and PXR, leading to inhibition of CYP7A1 [119,123,124]. In animals, under conditions of experimental cholestasis caused by ligation of the common bile duct, suppression of CYP27A1 activity and an increase in the relative proportion of CA were noted [125].

One of the key aspects of the pathogenesis of cholestatic liver diseases is the aging of biliary epithelial cells, which is expressed by morphological and molecular genetic changes, reflecting a decrease in the ability to proliferate and functional activity, and an increased tendency to apoptosis and tumor transformation [126]. Aging cholangiocytes secrete proinflammatory cytokines and chemokines that activate hepatic stellate cells, which promotes fibrogenesis and increases the risk of tumor growth [127]. Such changes may partly reflect the general aging process, and also be due to the effects of lipopolysaccharide and other damaging factors, particularly, carcinogens [126,128]. One of the manifestations of dystrophic changes in the biliary epithelium may be a decrease in the secretion of bicarbonates, which inhibit the penetration of BAs to the cell surface [129]. In primary biliary cholangitis, the expression of the anion exchanger AE2 and the secretion of bicarbonate are reduced in cholangiocytes [130]. Animal models show a decrease in bicarbonate secretion, corroborating laboratory and histological features of primary biliary cholangitis, including the appearance of anti-mitochondrial autoantibodies [131]. Mutations in the CFTR gene, against the background of which the secretion of bicarbonate is impaired, can also contribute to the development of cholestasis [132].

Cytokines can also have a significant effect on BA metabolism in hepatocytes. TNFα, produced by Kupffer cells, through interaction with the TNF receptor, activates the MAPK/JNK cascade, which leads to phosphorylation of HNF4α and suppression of the transcription of the CYP7A1 and CYP8B1 genes [133]. At the same time, the profibrogenic cytokine TGFβ1 produced during inflammatory reactions activates the promoter of CYP7A1 [134] CYP7A1 transcription in cholestatic diseases also suppresses insulin (signaling of the receptor which enhances BA) and the hepatocyte growth factor produced during inflammation. These cellular signaling pathways can be considered as an epigenetic mechanism for the protection of cells from BA overload in cholestasis [123,135].

The functional state of ABST determines the degree of absorption of BAs in the intestine and is likely to contribute to the course of cholestatic diseases. A study using cholescintigraphy with a taurine-conjugated 75Se-homocholic acid (75SeHCAT) showed that in primary biliary cholangitis, the absorption of BAs in the ileum is increased, which probably contributes to additional damage to liver cells, and with the appointment of UDCA, the degree of absorption of BAs decreased [136]. Preclinical studies have shown that FXR and PPAR agonists, ASBT antagonists, and a UDCA derivative, nor-UDCA, are promising for the treatment of primary sclerosing cholangitis [137,138].

In pediatric practice, progressing familial intrahepatic cholestasis syndromes 2, 3, and 5 (PFIC 2/3/5) are classic examples of cholestatic syndromes reflecting hereditary defects in BA transport. PFIC 2 occurs due to the low activity of BSEP; it is characterized by the low activity of β-glutamyl transpeptidase in the blood clot, which is involved in the export of BAs to bile. PFIC 3 occurs due to the low activity of MDR3 and a deficiency of phospholipids in bile, as a result of which BAs free from micelles attack the cells of the biliary epithelium, and crystallization of cholesterol is also observed. PFIC 5 is mediated by FXR inactivation with secondary suppression of BSEP and is characterized by the development of severe cholestasis [139]. The cause of the development of cholestatic syndrome in cystic fibrosis is a violation of the secretion of bicarbonate, as well as an obstructive component [140]. Regardless of the origin, cholestatic syndromes in infancy can lead to the common outcome of inflammatory cholangiopathy and extrahepatic biliary atresia [141].

In chronic cholestatic liver diseases, the risk of developing hepatocellular and cholangiocellular carcinoma increases, which is probably associated with the activation of proliferative processes under the influence of BA [142]. The damage caused by DCA has been shown, in an animal model, to stimulate the secretion of proinflammatory cytokines and protumorigenic factors by stellate cells. Thus, the so-called secretory phenotype associated with aging is realized. Experiments on animals have shown that DCA largely predisposes to hepatocellular carcinoma development [113].

In mouse models, it has been shown that tauro-CA and S1PR2 can promote the development of cholangiocellular cancer through the activation of EGFR, the ERK1/2/Akt-NF-κB signaling cascade, and COX-2. The interaction of conjugated bile acids with S1PR2 promotes the invasive growth of cholangiocarcinoma in the cell line [143]. The protumorigenic effect of DCA has also been shown [144]. Impairment of FXR function has also been shown to contribute to the spontaneous development of liver tumors [145].

BAs play an essential role in the pathogenesis of non-alcoholic fatty liver disease; however, many aspects of this phenomenon remain debated. It is assumed that the defect in the MAFG gene increases the relative content of CA and the absorption of cholesterol and BAs in the intestine, which contributes to the development of dyslipidemia [146].

Clinical studies (in particular, PIVENS and FLINT) have shown that the use of nuclear receptor agonists can lead to multidirectional effects, including undesirable ones. There is a paradoxical discrepancy in the indicators of steatosis, inflammation, fibrosis, and the development of insulin resistance, dyslipidemia, and obesity. Achievement of the desired effect may likely require the development of tissue-specific ligands or partial agonists of nuclear receptors [147].

## 9. Potential Role of Bile Acids in the Development of Lung Diseases

Gastroesophageal reflux and microaspiration can damage the airway epithelium and cause fibrosis. In the human epithelial cell line BEAS-2B, it was shown that LCA, even at a low concentration (>10 μmoL/L), causes death at a significantly higher concentration DCA, CDCA, and CA (30 μmoL /L). Exposure to BA resulted in a significant release of IL-8 and IL-6 from BEAS-2B cells [7]. BAs suppress the synthesis of inositol phosphate under the influence of acetylcholine in the smooth muscle cells of the respiratory tract rich in M3 receptors. A lung transplant revealed that BAs suppress the reactivity of bronchial smooth muscle cells in response to acetylcholine [148]. The negative effect of BAs can reduce bronchial clearance and contribute to the development of respiratory tract infections [149]. In cystic fibrosis, the effect of BA increases the tolerance to macrolides and polymyxin antibiotics. BA and CDCA promote the formation of *Pseudomonas aeruginosa* biofilms. Microbiome analysis of sputum samples in children with cystic fibrosis showed increased colonization of *P. aeruginosa* and other proteobacterial pathogens during bile aspiration [150]. The possible role of BAs entering the respiratory tract as part of gastroesophageal reflux in the pathogenesis of interstitial lung diseases is being studied [151].

## 10. Conclusions

Over the past decades, the interest of hepatologists in BAs has grown markedly [152]. The reason lies in the discovery of the role of BA in many different physiological processes. BAs comprise a group of molecular compounds with similar but not identical chemical structures. They have a variety of physical properties and different biological characteristics. While their most famous role is in the digestion and absorption of fat, they play an important role in several other functions. Table 1 summarizes the diverse biological processes in which BAs are involved.

In recent years, the importance of BAs has been demonstrated not only for the regulation of metabolism but also for the function and plasticity of immune cells. By interacting with receptors FXR, GPBAR1, VDR, and LXR, FAs can influence the course of inflammatory reactions. The relevance of these identified bile acids for inflammatory liver disease has not yet been studied, and future studies need to determine whether less common DCA or lithocholic acid derivatives come into contact with liver parenchymal and immune cells.

Moreover, BA signaling plays a vital role through receptor-dependent (FXR, VDR, GPBAR1, S1PR, M) and channel-mediated mechanisms in different cell types. In particular, with regard to the cardiac system, most studies have shown that BA signaling affects cardiac function, and cardiac dysfunction in liver disease is common. Further research in this area will allow for a more detailed description of the complex interactions between BA and their receptors in order to provide a pharmacological basis for the clinical treatment of related diseases.

Activation of the hypothalamus–pituitary–adrenal gland axis and increased release of cortisol are critical for a successful stress response, but this homeostatic mechanism is impaired in liver disease. In cirrhosis, an impaired adrenal response to adrenocorticotropic hormone increases mortality with impaired hemodynamics [41].

BAs are known to be secreted into the intestine, approximately 90% are reabsorbed in the portal system via the enterohepatic bloodstream and recirculated to hepatocytes [215]. However, in painful conditions, when reuptake is impaired, bile acids are released into the circulation, which can cause various pathological effects. Recent studies have shown that bile acids and bile acid signaling can influence a variety of neuropathological conditions.

The composition of the human gut microbiota can vary with diet, age, antibiotics, and disease. Bile acids appear to be the main regulator of the gut microbiota. In connection with a study by Kakiyama et al. a link has been suggested between liver health, fecal bile acid concentration, and gut microbiota composition [120]. In this study, fecal bile acid levels and community structure of the microbiome, as determined by quantification of the 16S ribosomal gene, were compared with control patients and patients with early and advanced cirrhosis. As cirrhosis progresses, it has been found that the bacterial dysbiosis seen in cirrhosis [216] is associated with low levels of bile acids entering the intestine [120]. This dysbiosis was characterized by a significant reduction in the number of Gram-positive members of the normal microbiota, such as *Blautia, Rumminococcaceae* [217]. An increase in pro-inflammatory and potentially pathogenic taxa of *Enterobacteriaceae* with the development of cirrhosis has been observed in patients with cirrhosis and decreased fecal BAs [120]. Thus, the size and composition of the bile acid pool appear to be important factors in the regulation of the structure of the human intestinal microbial community.

Due to their amphipathic characteristics, bile acids can behave like detergent molecules, which in many cases is the main cause of bile acid damage when they accumulate in the liver and other organs [218]. Elevated intracellular bile acid concentrations, such as those found in cholestasis, are associated with oxidative stress and apoptosis in both adult and fetal liver.

Finally, there is a link between bile acids and cell proliferation. Several types of bile acids have been shown to modulate DNA synthesis during liver regeneration after partial hepatectomy in rodents, and the regenerative process depends on bile acid signaling through the nuclear receptor FXR. The teratogenic [219] and carcinogenic [220] effects of more hydrophobic bile acids have been reported. Thus, bile acids have been suggested to play a role in the etiology of cancer at various sites—the colon, esophagus, or even non-digestive tissues such as the breast [221]. Moreover, it has recently been shown that mice lacking FXR spontaneously develop liver tumors [222].

Thus, the studies carried out in the past three decades have convincingly shown that the role of BAs is not limited to their participation only in digestion processes. Their role in various pathological processes is obvious, both as an etiological factor and as mediators of individual links of pathogenesis. However, based on the physicochemical and biological characteristics of BAs, it can be assumed that the list of their participation in pathological processes is far from complete and will be supplemented with the accumulation of scientific facts. These facts will make it possible to expand the indications for their use in clinical practice both in the form of independent drugs and in combination with other drugs acting at the cellular level or manifesting their effects at the molecular level through receptor-mediated cell mechanisms. There is no doubt that as knowledge of the physiological role of bile acids in the human body deepens, new concepts will appear that explain the reasons for the emergence and formation of a number of pathological processes, which are still unclear until today [223].

## Figures and Tables

**Table 1:** The role of bile acids in biological processes associated with the development of diseases.

BA Type	Effect	Probable Mechanism	Ref.
*Cardiovascular effects*			
DCA, LCA in vitro	Mesenterial arterial dilatation	Endothelial S1PR2 stimulation, ↑ Ca^2+^ intracellular concentration and ↓ NO production	[153,154]
BA (not specified) or CDCA in serum	↓ in mean arterial pressure and peripheral vascular resistance in cirrhosis. Could be involved in splanchnic hyperaemia and hyperdynamic syndrome	Endothelial FXR stimulation with ↑ eNOS and ↓ endothelin-1 and angiotensin-II receptor expression; ↓ vascular response to noradrenaline with DCA being the most potent inhibitor	[155,156,157,158,159,160,161]
Fasting BAs level in serum	Reversible association with atherosclerosis severity and the presence and severity of coronary artery disease, especially myocardial infarction	TGR5 stimulation with anti-inflammatory effect. Excess cholesterol excretion by secreting large amounts of BA into intestine. Activation of FXR (in animal models)	[162,163,164]
CDCA derivatives (oral administration)	Significantly ↓ aortic plaque formation and ↓ aortic expression of inflammatory factors (IL-6, IL-1, etc.) in apolipoprotein E-deficiency	Activation of FXR	[165]
Elevated serum BAs level in cirrhosis	Cirrhotic cardiomyopathy	Reduced fluidity of the myocardial membrane, resulting in adrenergic dysfunction and the inability to produce cAMP; ↓ myocardium contractility, apoptosis of cardiomyocytes, promoting myocardial ischemia/reperfusion injury, ↑ production of NO mediated by intracellular Ca^2+^ signaling	[161,166]
CA in cirrhosis/DCA and LCA in vitro and portal blood	Bradycardia	Altered cardiac membrane fluidity and decreased beta-adrenergic receptor signalling. DCA and LCA act as muscarinic antagonists	[166,167,168,169]
Non-UDCA/UDCA ratio in serum	Independent predictor of atrial fibrillation	↑ portion of non-UDCA can change slow inward Na^+^ and Ca^2+^ currents and outward K^+^ currents, ↓ the duration of the action potential in cardiomyocytes predisposing to re-entry type arrythmia	[38,170,171]
Supraphysiological tauro-CA concentration in vitro and in intestine	A role in progressing of heart failure	Depolarization of the resting potential and inducing posterior depolarization of cells (reduced contractility and pacemaker activity). Decrease protein expression in heart tissue.	[172,173,174]
↑ ratio of secondary BAs to primary BAs in intestine	--	Indirect influence of the intestinal flora on the severity of HF hydrophobic BAs significantly alter mitochondrial bioenergetics	[37,172,175]
*Intestinal microbiota modifications*			
Primary bile acids per os	Prevention of in overgrowth of aerobic and anaerobic bacteria in the ileum and cecum and of bacterial translocation	FXRα activation resulting in up-regulation of genes involved in mucosal defense in the ileum. Direct antimicrobial effects in high concentration of conjugated BAs	[176,177]
↑ CA per os	↑ in *Firmicutes*, especially groups capable of 7α-dehydroxylation, and ↓ of *Bacteroidetes*	Due to sustaining of 7α-dehydroxylating bacteria and antagonistic effect on other bacterial communities (↑ production of an antimicrobial compounds by these members, or use of BAs as an electron acceptor in metabolic pathways providing a net energy gain)/BA induce (*bai*) operon	[76,178,179]
Primary bile acids (tauro-CA) in intestine	Recovery of microbiota after dysbiosis induced by antibiotics or toxins	Provide homing signals to gut bacteria and promote germination of spores. This mechanism can be exploited by pathogens such as *Clostridium difficile*, germinating into a vegetative form	[120,180]
More hydrophobic bile acids (having two rather than three hydroxy groups) in intestine	Inhibition of bacterial overgrowth	Impair the membrane integrity. FXR activates genes involved in enteric protection (*ANG1*, *iNOS*). Induce ERK 1/2 pathway which activates the VDR and the synthesis of antimicrobial peptides cathelicidins	[85,181,182]
*Metabolic effects*			
Dehydroxylated Bas in intestine	Significant reduction in host weight gain, plasma cholesterol, and liver triglycerides	Activation transcription of key genes involved in lipid metabolism (PPARγ, ANGPTL 4), cholesterol metabolism (ABCG 5/8), gastrointestinal homeostasis (REG 3γ), and circadian rhythm (DBP, PER1/2) in the liver or small intestine (probably through the FXRα activation)	[183]
BAs in intestine	↑ energy expenditure in brown adipose tissue and muscle	TGR5 can stimulate glucagon-like protein 1, improving glucose tolerance and activating thyroid hormone	[184,185]
	Improve glucose homeostasis and triglyceride control aspects of metabolic syndrome in animal models	Activation of FXRα	[186]
Decreased concentration of BAs in intestine (in acid-binding resins application)	Stimulate the conversion of cholesterol to bile acids	Activation of FXRα	[187]
BAs or their synthetic derivatives per os	↓ serum triglycerides and total cholesterol, inhibition of the atherosclerosis in a dose-dependent manner.	Activation of FXRα	[188]
CA and other FXRα agonists per os	↑ serum HDL and phospholipids but decreased ApoA-1 (controversial results)	FXRα induction leading to ↓ SREBP1c (through SHP and LXRα/LXRβ) and triglyceride synthesis and VLDL level. FXRα induction leading to SR-B1 activation ↑ total and serum HDL cholesterol suggesting that reverse cholesterol transport is disrupted. Probable role of epigenetic mechanisms.	[189,190,191]
*Role in fatty liver disease*			
Glycine-conjugated BAs	Positive correlation with macrovesicular steatosis score	Inhibition of CYP8B1 and stimulation of CYP7B1 expression in NASH livers (suggests a shift to alternative pathway of BAs synthesis)	[192]
Oral CA and UDCA	Improvement in hepatic steatosis	Under the stidy	[193]
*Role in cholestatic liver disease*			
Accumulation of hydrophobic bile acids DCA and CDCA in the liver	Cholestatic liver injury	Membrane desorganisation stimulates production of reactive oxygen species and activation of NF-κB	[194]
Total BAs in fetal serum in intrahepatic cholestasis of pregnancy	Association with ventricular arrhythmia in pregnant women	Abnormal ventricular repolarization	[195]
Oral UDCA/tauro-UDCA	Protection of cholangiocytes against cytotoxicity of hydrophobic bile acids, stimulation of hepatobiliary secretion, and protection of hepatocytes against BAs- induced apoptosis	Modulation of the composition of mixed phospholipid-rich micelles, possibly, decrease in the concentration of hydrophobic bile acids in the cholangiocytes. Stimulation of Ca(2+)- and protein kinase C-alpha-dependent mechanisms and/or activation of p38 (MAPK) and extracellular signal-regulated kinases (ERK) resulting in insertion of transporter molecules (BSEP, MRP2) into the canalicular membrane and NTCP into the basolateral membrane. Inhibition of mitochondrial membrane permeability transition, and possibly, stimulation of a survival pathway. Counteraction with the action of toxic BAs reduces endoplasmic reticulum stress. TUDC initiates differentiation of multipotent mesenchymal stem cells. α_5_β_1_ integrins probably serve as sensors for TUDC with the downstream activation of focal adhesion kinase, c-SRC, the epidermal growth factor receptor and activation of the mitogen-activated protein kinases, ERKs and p38.	[196,197]
Obeticholic acid (a selective potent FXR agonist, structural CDCA analog)	Anticholestatic and antifibrotic properties in primary biliary cholangitis not responding to first-line treatment; ↓ portal pressure without a ↓ in mean arterial pressure. Protective cardiopulmonary effect in both cholestatic cirrhotic rat models. Ileal barrier function improvement, reduced bacterial translocation.	FXR activation with decreased BAs synthesis. Increased intrahepatic eNOS activity. Interaction with Kupffer cells and expression of IL-1 and TNFα with concomitant repression of CYP7A1 in hepatocytes.	[198,199,200,201,202,203]
*Role in carcinogenesis*			
Accumulation DCA and CDCA in the liver	Hepatocellular carcinoma development	Stimulates production of reactive oxygen species and activation of RAS and NF-κB, proinflammatory or tumorogenic factors in the liver with subsequent downregulating of FXR and SHP—an important tumor suppressor.	[204,205,206]
↑ levels of DCA, LCA in stool	Pro-carcinogenic potential in the colon	Generation of cancer stem cells probably through Wnt/β-catenin signaling	[207]
Tauro-CA in the colon	Pro-carcinogenic potential in the colon	Genotoxic effects are under investigation. Metabolism of taurine conjugated BAs by gut microbes generates a genotoxic hydrogen sulfide	[208]
BAs in duodenal refluctate	Esophageal dysplasia, squamous cell carcinoma and adenocarcinoma	Expression of COX2 and p53 in esophageal proliferating cells	[209,210]
*Bowel diseases*			
Altered colonic BA (shift to CA and tauro-CA) in colon after antibiotic treatment	Association with several disease states, including recurrent *C. difficile* infection (with cases of CDI pouchitis)	A permissive environment in which the bacterium may thrive stimulate germination of *C. difficile* spores. CDCA, LCA, and UDCA inhibit germination of spores	[211,212]
*Esophageal diseases*			
Oral UDCA	May protect against DNA damage induced by hydrophobic bile acids such as DCA in the metaplastic mucosa of patients with Barrett’s esophagus	UDCA counters the DNA damaging effects of DCA	[213]
*Lung disease*			
Repeated microaspiration of CDCA, DCA, and LCA	Fibrotic changes in alveolar wall	Stimulation of fibrogenic mediator expression and activating TGF-β1/SMAD3 signaling and FXR	[150]
BAs in the lung tissue in cystic fibrosis	Association with inflammation and restructuring of the lung microbiota with a dominance of *Proteobacteria*	Tissue damage, bactericidal effect.	[214]

Abbreviation: ABCG 5/8—ATP-binding cassette sub-family G member 5/5; ANGPTL 4—angiopoietin-related protein 4; *ANG1*—angiogenin gene 1; BA—bile acids, BSEP—bile salt exporting pump, CA—cholic acid; cAMP—cyclic adenosine monophosphate; CDCA—chenodeoxycholic acid; COX2—cyclooxygenase-2; c-SRC—proto-oncogene; CYP7B1—cytochrome P450 family 7 subfamily B member 1; CYP8B1—cytochrome P450, family 8, subfamily B, polypeptide 1; DBP—D-box binding PAR BZIP transcription factor; DCA—deoxycholic acid; DNA—deoxyribonucleic acid; ERK—1/2 extracellular signal-regulated kinase 1/2; eNOS—endothelial nitric oxide synthase; *iNOS*—inducible nitric oxide synthase; HF—heart failure; FXR—farnesoid X receptor; IL-1—interleukin 1; IL-6—interleukin 6; LCA—lithocholic acid; LXR—liver X receptor; MAPK—mitogen-activated protein kinase; MRP2—multidrug resistance-associated protein 2; NASH—non-alcoholic steatohepatitis; NF-κB—nuclear factor-kappa B; NO—nitric oxide; NTCP—Na^+^-taurocholate cotransporting polypeptide; PER1/2—period circadian protein homolog 1/2; PPARγ—peroxisome proliferator-activated receptor γ; RAS—from “Rat sarcoma virus”, is a family of related proteins; REG 3γ—regenerating islet-derived protein 3γ; SHP—small heterodimer partner; SMAD3—mothers against decapentaplegic homolog 3; S1PR2—sphingosine-1-phosphate receptor 2; TGR5—G protein-coupled bile acid receptor 5; TGF-β1—transforming growth factor-beta 1; TNFα—tumor necrosis factor-alpha; TUDC—tauroursodeoxycholate; UDCA—ursodeoxycholic acid; VDR—vitamin D receptor. Designation: ↑—increase; ↓—decrease.

## Data Availability

This is a review paper that collected from public data listed in the “Reference” and from open-access web-source PubMed.

## References

[1] Russell D.W. (2003). The Enzymes, Regulation, and Genetics of Bile Acid Synthesis. Annu. Rev. Biochem..

[2] Evangelakos I., Heeren J., Verkade E., Kuipers F. (2021). Role of Bile Acids in Inflammatory Liver Diseases. Semin. Immunopathol..

[3] Khurana S., Raufman J.P., Pallone T.L. (2011). Bile Acids Regulate Cardiovascular Function. Clin. Transl. Sci..

[4] Lieu T., Jayaweera G., Bunnett N.W. (2014). GPBA: A GPCR for Bile Acids and an Emerging Therapeutic Target for Disorders of Digestion and Sensation: GPBA (TGR5) Bile Acid Receptor. Br. J. Pharmacol..

[5] Ackerman H.D., Gerhard G.S. (2016). Bile Acids in Neurodegenerative Disorders. Front. Aging Neurosci..

[6] Fiorucci S., Distrutti E. (2015). Bile Acid-Activated Receptors, Intestinal Microbiota, and the Treatment of Metabolic Disorders. Trends Mol. Med..

[7] Aldhahrani A., Verdon B., Ward C., Pearson J. (2017). Effects of Bile Acids on Human Airway Epithelial Cells: Implications for Aerodigestive Diseases. ERJ Open Res..

[8] Nasr A.O., Robb W., Walsh T.N. (2013). Review article; duodeno-gastro-esophageal reflux combined and isolated. Am. Med. J..

[9] Brodlie M., Aseeri A., Lordan J.L., Robertson A.G.N., McKean M.C., Corris P.A., Griffin S.M., Manning N.J., Pearson J.P., Ward C. (2015). Bile Acid Aspiration in People with Cystic Fibrosis before and after Lung Transplantation. Eur. Respir. J..

[10] Biagioli M., Fiorucci S. (2021). Bile Acid Activated Receptors: Integrating Immune and Metabolic Regulation in Non-Alcoholic Fatty Liver Disease. Liver Res..

[11] Keitel V., Donner M., Winandy S., Kubitz R., Häussinger D. (2008). Expression and Function of the Bile Acid Receptor TGR5 in Kupffer Cells. Biochem. Biophys. Res. Commun..

[12] Fiorucci S., Biagioli M., Zampella A., Distrutti E. (2018). Bile Acids Activated Receptors Regulate Innate Immunity. Front. Immunol..

[13] Lou G., Ma X., Fu X., Meng Z., Zhang W., Wang Y.D., Van Ness C., Yu D., Xu R., Huang W. (2014). GPBAR1/TGR5 Mediates Bile Acid-Induced Cytokine Expression in Murine Kupffer Cells. PLoS ONE.

[14] Biagioli M., Carino A., Cipriani S., Francisci D., Marchianò S., Scarpelli P., Sorcini D., Zampella A., Fiorucci S. (2017). The Bile Acid Receptor GPBAR1 Regulates the M1/M2 Phenotype of Intestinal Macrophages and Activation of GPBAR1 Rescues Mice from Murine Colitis. J. Immunol..

[15] Keitel V., Reinehr R., Gatsios P., Rupprecht C., Görg B., Selbach O., Häussinger D., Kubitz R. (2007). The G-Protein Coupled Bile Salt Receptor TGR5 Is Expressed in Liver Sinusoidal Endothelial Cells. Hepatology.

[16] Spatz M., Ciocan D., Merlen G., Rainteau D., Humbert L., Gomes-Rochette N., Hugot C., Trainel N., Mercier-Nomé F., Domenichini S. (2021). Bile Acid-Receptor TGR5 Deficiency Worsens Liver Injury in Alcohol-Fed Mice by Inducing Intestinal Microbiota Dysbiosis. JHEP Rep..

[17] Jourdainne V., Péan N., Doignon I., Humbert L., Rainteau D., Tordjmann T. (2015). The Bile Acid Receptor TGR5 and Liver Regeneration. Dig. Dis..

[18] Baghdasaryan A., Jha P., Müller M., Agostinelli L., Saccomanno S., Auer N., Deutschmann A., Zöhrer C., Auwerx J., Marzioni M. (2014). P389 protective role of membrane bile acid receptor tgr5 (gpbar1) in ddc-induced sclerosing cholangitis in mice. J. Hepatol..

[19] Yang Z.H., Liu F., Zhu X.R., Suo F.Y., Jia Z.J., Yao S.K. (2021). Altered Profiles of Fecal Bile Acids Correlate with Gut Microbiota and Inflammatory Responses in Patients with Ulcerative Colitis. World J. Gastroenterol..

[20] Wahlström A., Sayin S.I., Marschall H.U., Bäckhed F. (2016). Intestinal Crosstalk between Bile Acids and Microbiota and Its Impact on Host Metabolism. Cell. Metab..

[21] Hang S., Paik D., Yao L., Kim E., Trinath J., Lu J., Ha S., Nelson B.N., Kelly S.P., Wu L. (2019). Bile Acid Metabolites Control TH17 and Treg Cell Differentiation. Nature.

[22] Campbell C., McKenney P.T., Konstantinovsky D., Isaeva O.I., Schizas M., Verter J., Mai C., Jin W.B., Guo C.J., Violante S. (2020). Bacterial Metabolism of Bile Acids Promotes Generation of Peripheral Regulatory T Cells. Nature.

[23] Ross R. (1999). Atherosclerosis—An Inflammatory Disease. N. Engl. J. Med..

[24] Glass C.K., Witztum J.L. (2001). Atherosclerosis. The Road Ahead. Cell.

[25] González N.A., Castrillo A. (2011). Liver X Receptors as Regulators of Macrophage Inflammatory and Metabolic Pathways. Biochim. Biophys. Acta BBA Mol. Basis Dis..

[26] Ljubuncic P., Said O., Ehrlich Y., Meddings J.B., Shaffer E.A., Bomzon A. (2000). On the in Vitro Vasoactivity of Bile Acids: The in Vitro Vasoactivity of Bile Acids. Br. J. Pharmacol..

[27] Pols T.W.H., Nomura M., Harach T., Lo Sasso G., Oosterveer M.H., Thomas C., Rizzo G., Gioiello A., Adorini L., Pellicciari R. (2011). TGR5 Activation Inhibits Atherosclerosis by Reducing Macrophage Inflammation and Lipid Loading. Cell Metab..

[28] Barchetta I. (2021). Expression of TGR5 in Adipose Tissue in Relation to Metabolic Impairment and Adipose Tissue Dysfunction in Human Obesity. Metab. Target Organ Damage.

[29] Hageman J., Herrema H., Groen A.K., Kuipers F. (2010). A Role of the Bile Salt Receptor FXR in Atherosclerosis. Arterioscler. Thromb. Vasc. Biol..

[30] Vasavan T., Ferraro E., Ibrahim E., Dixon P., Gorelik J., Williamson C. (2018). Heart and Bile Acids–Clinical Consequences of Altered Bile Acid Metabolism. Biochim. Biophys. Acta BBA Mol. Basis Dis..

[31] Zhu L., Chen Z., Han K., Zhao Y., Li Y., Li D., Wang X., Li X., Sun S., Lin F. (2020). Correlation between Mitochondrial Dysfunction, Cardiovascular Diseases, and Traditional Chinese Medicine. Evid. Based Complement. Alternat. Med..

[32] Mu Y.P., Peng S.Y. (1997). Relation between Bile Acids and Myocardial Damage in Obstructive Jaundice. World J. Gastroenterol..

[33] Joubert P. (1978). Cholic acid and the heart: In vitro studies of the effect on heart rate and myocardial contractility in the rat. Clin. Exp. Pharmacol. Physiol..

[34] Pu J., Yuan A., Shan P., Gao E., Wang X., Wang Y., Lau W.B., Koch W., Ma X.L., He B. (2013). Cardiomyocyte-Expressed Farnesoid-X-Receptor Is a Novel Apoptosis Mediator and Contributes to Myocardial Ischaemia/Reperfusion Injury. Eur. Heart J..

[35] Halestrap A.P., Pasdois P. (2009). The Role of the Mitochondrial Permeability Transition Pore in Heart Disease. Biochim. Biophys. Acta.

[36] Desai M.S., Mathur B., Eblimit Z., Vasquez H., Taegtmeyer H., Karpen S.J., Penny D.J., Moore D.D., Anakk S. (2017). Bile Acid Excess Induces Cardiomyopathy and Metabolic Dysfunctions in the Heart. Hepatology.

[37] Ferreira M., Coxito P.M., Sardão V.A., Palmeira C.M., Oliveira P.J. (2005). Bile Acids Are Toxic for Isolated Cardiac Mitochondria: A Possible Cause for Hepatic-Derived Cardiomyopathies?. Cardiovasc. Toxicol..

[38] Rainer P.P., Primessnig U., Harenkamp S., Doleschal B., Wallner M., Fauler G., Stojakovic T., Wachter R., Yates A., Groschner K. (2013). Bile Acids Induce Arrhythmias in Human Atrial Myocardium—Implications for Altered Serum Bile Acid Composition in Patients with Atrial Fibrillation. Heart.

[39] Liu L., Panzitt K., Racedo S., Wagner M., Platzer W., Zaufel A., Theiler-Schwetz V., Obermayer-Pietsch B., Müller H., Höfler G. (2019). Bile Acids Increase Steroidogenesis in Cholemic Mice and Induce Cortisol Secretion in Adrenocortical H295R Cells via S1 PR 2, ERK and SF–1. Liver Int..

[40] Frey F.J. (2006). Impaired 11β-Hydroxysteroid Dehydrogenase Contributes to Renal Sodium Avidity in Cirrhosis: Hypothesis or Fact?. Hepatology.

[41] McNeilly A.D., Macfarlane D.P., O’Flaherty E., Livingstone D.E., Mitić T., McConnell K.M., McKenzie S.M., Davies E., Reynolds R.M., Thiesson H.C. (2010). Bile Acids Modulate Glucocorticoid Metabolism and the Hypothalamic–Pituitary–Adrenal Axis in Obstructive Jaundice. J. Hepatol..

[42] Rose A.J., Díaz M.B., Reimann A., Klement J., Walcher T., Krones-Herzig A., Strobel O., Werner J., Peters A., Kleyman A. (2011). Molecular Control of Systemic Bile Acid Homeostasis by the Liver Glucocorticoid Receptor. Cell Metab..

[43] Schmidt D.R., Schmidt S., Holmstrom S.R., Makishima M., Yu R.T., Cummins C.L., Mangelsdorf D.J., Kliewer S.A. (2011). AKR1B7 Is Induced by the Farnesoid X Receptor and Metabolizes Bile Acids. J. Biol. Chem..

[44] Huang F., Wang T., Lan Y., Yang L., Pan W., Zhu Y., Lv B., Wei Y., Shi H., Wu H. (2015). Deletion of Mouse FXR Gene Disturbs Multiple Neurotransmitter Systems and Alters Neurobehavior. Front. Behav. Neurosci..

[45] Di Somma C., Scarano E., Barrea L., Zhukouskaya V., Savastano S., Mele C., Scacchi M., Aimaretti G., Colao A., Marzullo P. (2017). Vitamin D and Neurological Diseases: An Endocrine View. Int. J. Mol. Sci..

[46] Eyles D.W., Smith S., Kinobe R., Hewison M., McGrath J.J. (2005). Distribution of the Vitamin D Receptor and 1α-Hydroxylase in Human Brain. J. Chem. Neuroanat..

[47] Buell J.S., Dawson-Hughes B. (2008). Vitamin D and Neurocognitive Dysfunction: Preventing “D”Ecline?. Mol. Aspects Med..

[48] Grant S.M., DeMorrow S. (2020). Bile Acid Signaling in Neurodegenerative and Neurological Disorders. Int. J. Mol. Sci..

[49] Ikura T., Ito N. (2016). Crystal Structure of the Vitamin D Receptor Ligand-Binding Domain with Lithocholic Acids. Vitamins and Hormones.

[50] Kim E.Y., Lee J.M. (2022). Transcriptional Regulation of Hepatic Autophagy by Nuclear Receptors. Cells.

[51] Li R., Guo E., Yang J., Li A., Yang Y., Liu S., Liu A., Jiang X. (2017). 1,25(OH)_2_D_3_ Attenuates Hepatic Steatosis by Inducing Autophagy in Mice: 1,25(OH)_2_D_3_ Attenuates Hepatic Steatosis. Obesity.

[52] Yuan F., Xu Y., You K., Zhang J., Yang F., Li Y. (2021). Calcitriol Alleviates Ethanol-Induced Hepatotoxicity via AMPK/MTOR-Mediated Autophagy. Arch. Biochem. Biophys..

[53] D’Aldebert E., Biyeyeme Bi Mve M., Mergey M., Wendum D., Firrincieli D., Coilly A., Fouassier L., Corpechot C., Poupon R., Housset C. (2009). Bile Salts Control the Antimicrobial Peptide Cathelicidin Through Nuclear Receptors in the Human Biliary Epithelium. Gastroenterology.

[54] Han S., Chiang J.Y.L. (2009). Mechanism of Vitamin D Receptor Inhibition of Cholesterol 7α-Hydroxylase Gene Transcription in Human Hepatocytes. Drug Metab. Dispos..

[55] Reddy D.S. (2010). Neurosteroids: Endogenous Role in the Human Brain and Therapeutic Potentials. Prog. Brain Res..

[56] Keitel V., Görg B., Bidmon H.J., Zemtsova I., Spomer L., Zilles K., Häussinger D. (2010). The Bile Acid Receptor TGR5 (GPBAR-1) Acts as a Neurosteroid Receptor in Brain. Glia.

[57] Schubring S.R., Fleischer W., Lin J.S., Haas H.L., Sergeeva O.A. (2012). The Bile Steroid Chenodeoxycholate Is a Potent Antagonist at NMDA and GABAA Receptors. Neurosci. Lett..

[58] Yanovsky Y., Schubring S.R., Yao Q., Zhao Y., Li S., May A., Haas H.L., Lin J.-S., Sergeeva O.A. (2012). Waking Action of Ursodeoxycholic Acid (UDCA) Involves Histamine and GABAA Receptor Block. PLoS ONE.

[59] Silva S.L., Vaz A.R., Diógenes M.J., van Rooijen N., Sebastião A.M., Fernandes A., Silva R.F.M., Brites D. (2012). Neuritic Growth Impairment and Cell Death by Unconjugated Bilirubin Is Mediated by NO and Glutamate, Modulated by Microglia, and Prevented by Glycoursodeoxycholic Acid and Interleukin-10. Neuropharmacology.

[60] Palmela I., Correia L., Silva R.F.M., Sasaki H., Kim K.S., Brites D., Brito M.A. (2015). Hydrophilic Bile Acids Protect Human Blood-Brain Barrier Endothelial Cells from Disruption by Unconjugated Bilirubin: An in Vitro Study. Front. Neurosci..

[61] Quinn M., McMillin M., Galindo C., Frampton G., Pae H.Y., DeMorrow S. (2014). Bile Acids Permeabilize the Blood Brain Barrier after Bile Duct Ligation in Rats via Rac1-Dependent Mechanisms. Dig. Liver Dis..

[62] Sun D., Gu G., Wang J., Chai Y., Fan Y., Yang M., Xu X., Gao W., Li F., Yin D. (2017). Administration of Tauroursodeoxycholic Acid Attenuates Early Brain Injury via Akt Pathway Activation. Front. Cell. Neurosci..

[63] Romero-Ramírez L., Nieto-Sampedro M., Yanguas-Casás N. (2017). Tauroursodeoxycholic Acid: More than Just a Neuroprotective Bile Conjugate. Neural Regen. Res..

[64] Payne T., Sassani M., Buckley E., Moll S., Anton A., Appleby M., Maru S., Taylor R., McNeill A., Hoggard N. (2020). Ursodeoxycholic Acid as a Novel Disease-Modifying Treatment for Parkinson’s Disease: Protocol for a Two-Centre, Randomised, Double-Blind, Placebo-Controlled Trial, The “UP” Study. BMJ Open.

[65] Higashi T., Watanabe S., Tomaru K., Yamazaki W., Yoshizawa K., Ogawa S., Nagao H., Minato K., Maekawa M., Mano N. (2017). Unconjugated Bile Acids in Rat Brain: Analytical Method Based on LC/ESI-MS/MS with Chemical Derivatization and Estimation of Their Origin by Comparison to Serum Levels. Steroids.

[66] McMillin M., Frampton G., Tobin R., Dusio G., Smith J., Shin H., Newell-Rogers K., Grant S., DeMorrow S. (2015). TGR5 Signaling Reduces Neuroinflammation during Hepatic Encephalopathy. J. Neurochem..

[67] Nizamutdinov D., DeMorrow S., McMillin M., Kain J., Mukherjee S., Zeitouni S., Frampton G., Bricker P.C.S., Hurst J., Shapiro L.A. (2017). Hepatic Alterations Are Accompanied by Changes to Bile Acid Transporter-Expressing Neurons in the Hypothalamus after Traumatic Brain Injury. Sci. Rep..

[68] Klaassen C.D., Aleksunes L.M. (2010). Xenobiotic, Bile Acid, and Cholesterol Transporters: Function and Regulation. Pharmacol. Rev..

[69] Tripodi V., Contin M., Fernández M.A., Lemberg A. (2012). Bile Acids Content in Brain of Common Duct Ligated Rats. Annu. Hepatol..

[70] Mertens K.L., Kalsbeek A., Soeters M.R., Eggink H.M. (2017). Bile Acid Signaling Pathways from the Enterohepatic Circulation to the Central Nervous System. Front. Neurosci..

[71] Kremer A.E., Namer B., Bolier R., Fischer M.J., Oude Elferink R.P., Beuers U. (2015). Pathogenesis and Management of Pruritus in PBC and PSC. Dig. Dis..

[72] Krähenbühl S., Talos C., Fischer S., Reichen J. (1994). Toxicity of Bile Acids on the Electron Transport Chain of Isolated Rat Liver Mitochondria. Hepatology.

[73] Yang C., Jin C., Li X., Wang F., McKeehan W.L., Luo Y. (2012). Differential Specificity of Endocrine FGF19 and FGF21 to FGFR1 and FGFR4 in Complex with KLB. PLoS ONE.

[74] Kuhre R.E., Wewer Albrechtsen N.J., Larsen O., Jepsen S.L., Balk-Møller E., Andersen D.B., Deacon C.F., Schoonjans K., Reimann F., Gribble F.M. (2018). Bile Acids Are Important Direct and Indirect Regulators of the Secretion of Appetite- and Metabolism-Regulating Hormones from the Gut and Pancreas. Mol. Metab..

[75] Meaney S., Heverin M., Panzenboeck U., Ekström L., Axelsson M., Andersson U., Diczfalusy U., Pikuleva I., Wahren J., Sattler W. (2007). Novel Route for Elimination of Brain Oxysterols across the Blood-Brain Barrier: Conversion into 7α-Hydroxy-3-Oxo-4-Cholestenoic Acid. J. Lipid Res..

[76] Ridlon J.M., Kang D.J., Hylemon P.B., Bajaj J.S. (2014). Bile Acids and the Gut Microbiome. Curr. Opin. Gastroenterol..

[77] Yang H., Duan Z. (2016). Bile Acids and the Potential Role in Primary Biliary Cirrhosis. Digestion.

[78] Garmendia J., Frankel G., Crepin V.F. (2005). Enteropathogenic and Enterohemorrhagic *Escherichia Coli* Infections: Translocation, Translocation, Translocation. Infect. Immun..

[79] Begley M., Gahan C.G.M., Hill C. (2002). Bile Stress Response in *Listeria Monocytogenes* LO28: Adaptation, Cross-Protection, and Identification of Genetic Loci Involved in Bile Resistance. Appl. Environ. Microbiol..

[80] Prouty A.M., Gunn J.S. (2000). *Salmonella Enterica* Serovar Typhimurium Invasion Is Repressed in the Presence of Bile. Infect. Immun..

[81] Nickerson K.P., Chanin R.B., Sistrunk J.R., Rasko D.A., Fink P.J., Barry E.M., Nataro J.P., Faherty C.S. (2017). Analysis of Shigella Flexneri Resistance, Biofilm Formation, and Transcriptional Profile in Response to Bile Salts. Infect. Immun..

[82] Faherty C.S., Redman J.C., Rasko D.A., Barry E.M., Nataro J.P. (2012). Shigella Flexneri Effectors OspE1 and OspE2 Mediate Induced Adherence to the Colonic Epithelium Following Bile Salts Exposure: OspE1 and OspE2 Enhance Adherence to Epithelial Cells. Mol. Microbiol..

[83] Malik-Kale P., Parker C.T., Konkel M.E. (2008). Culture of *Campylobacter Jejuni* with Sodium Deoxycholate Induces Virulence Gene Expression. J. Bacteriol..

[84] Solheim M., Aakra Å., Vebø H., Snipen L., Nes I.F. (2007). Transcriptional Responses of *Enterococcus Faecalis* V583 to Bovine Bile and Sodium Dodecyl Sulfate. Appl. Environ. Microbiol..

[85] Urdaneta V., Casadesús J. (2017). Interactions between Bacteria and Bile Salts in the Gastrointestinal and Hepatobiliary Tracts. Front. Med..

[86] Francis M.B., Allen C.A., Shrestha R., Sorg J.A. (2013). Bile Acid Recognition by the Clostridium Difficile Germinant Receptor, CspC, Is Important for Establishing Infection. PLoS Pathog..

[87] Sorg J.A., Sonenshein A.L. (2010). Inhibiting the Initiation of *Clostridium Difficile* Spore Germination Using Analogs of Chenodeoxycholic Acid, a Bile Acid. J. Bacteriol..

[88] Watanabe M., Fukiya S., Yokota A. (2017). Comprehensive Evaluation of the Bactericidal Activities of Free Bile Acids in the Large Intestine of Humans and Rodents. J. Lipid Res..

[89] Sannasiddappa T.H., Lund P.A., Clarke S.R. (2017). In Vitro Antibacterial Activity of Unconjugated and Conjugated Bile Salts on Staphylococcus Aureus. Front. Microbiol..

[90] Prete R., Long S.L., Gallardo A.L., Gahan C.G., Corsetti A., Joyce S.A. (2020). Beneficial Bile Acid Metabolism from Lactobacillus Plantarum of Food Origin. Sci. Rep..

[91] Faillie J.L., Yu O.H., Yin H., Hillaire-Buys D., Barkun A., Azoulay L. (2016). Association of Bile Duct and Gallbladder Diseases with the Use of Incretin-Based Drugs in Patients with Type 2 Diabetes Mellitus. JAMA Intern. Med..

[92] Bernstein H., Bernstein C., Payne C.M., Dvorakova K., Garewal H. (2005). Bile Acids as Carcinogens in Human Gastrointestinal Cancers. Mutat. Res. Mutat. Res..

[93] Vavassori P., Mencarelli A., Renga B., Distrutti E., Fiorucci S. (2009). The Bile Acid Receptor FXR Is a Modulator of Intestinal Innate Immunity. J. Immunol..

[94] Camilleri M. (2014). Advances in Understanding of Bile Acid Diarrhea. Expert Rev. Gastroenterol. Hepatol..

[95] Camilleri M., Nadeau A., Tremaine W.J., Lamsam J., Burton D., Odunsi S., Sweetser S., Singh R. (2009). Measurement of Serum 7α-Hydroxy-4-Cholesten-3-One (or 7αC4), a Surrogate Test for Bile Acid Malabsorption in Health, Ileal Disease and Irritable Bowel Syndrome Using Liquid Chromatography-Tandem Mass Spectrometry. Neurogastroenterol. Motil..

[96] Farrugia A., Arasaradnam R. (2021). Bile Acid Diarrhoea: Pathophysiology, Diagnosis and Management. Frontline Gastroenterol..

[97] Li W.T., Luo Q.Q., Wang B., Chen X., Yan X.J., Qiu H.Y., Chen S.L. (2019). Bile Acids Induce Visceral Hypersensitivity *via* Mucosal Mast Cell–to–Nociceptor Signaling That Involves the Farnesoid X Receptor/Nerve Growth Factor/Transient Receptor Potential Vanilloid 1 Axis. FASEB J..

[98] Centuori S.M., Martinez J.D. (2014). Differential Regulation of EGFR–MAPK Signaling by Deoxycholic Acid (DCA) and Ursodeoxycholic Acid (UDCA) in Colon Cancer. Dig. Dis. Sci..

[99] Cook J.W., Kennaway E.L., Kennaway N.M. (1940). Production of Tumours in Mice by Deoxycholic Acid. Nature.

[100] Fu T., Coulter S., Yoshihara E., Oh T.G., Fang S., Cayabyab F., Zhu Q., Zhang T., Leblanc M., Liu S. (2019). FXR Regulates Intestinal Cancer Stem Cell Proliferation. Cell.

[101] Akare S., Martinez J.D. (2005). Bile Acid Induces Hydrophobicity-Dependent Membrane Alterations. Biochim. Biophys. Acta.

[102] Bernstein C., Holubec H., Bhattacharyya A.K., Nguyen H., Payne C.M., Zaitlin B., Bernstein H. (2011). Carcinogenicity of Deoxycholate, a Secondary Bile Acid. Arch. Toxicol..

[103] Kim E.K., Cho J.H., Kim E., Kim Y.J. (2017). Ursodeoxycholic Acid Inhibits the Proliferation of Colon Cancer Cells by Regulating Oxidative Stress and Cancer Stem-like Cell Growth. PLoS ONE.

[104] Kim Y.H., Kim J.H., Kim B.G., Lee K.L., Kim J.W., Koh S.J. (2019). Tauroursodeoxycholic Acid Attenuates Colitis-Associated Colon Cancer by Inhibiting Nuclear Factor KappaB Signaling: Effects of TUDCA in Colitis. J. Gastroenterol. Hepatol..

[105] Peng Y., Nie Y., Yu J., Wong C.C. (2021). Microbial Metabolites in Colorectal Cancer: Basic and Clinical Implications. Metabolites.

[106] Powell A.A., LaRUE J.M., Batta A.K., Martinez J.D. (2001). Bile Acid Hydrophobicity Is Correlated with Induction of Apoptosis and/or Growth Arrest in HCT116 Cells. Biochem. J..

[107] Sips F.L.P., Eggink H.M., Hilbers P.A.J., Soeters M.R., Groen A.K., van Riel N.A.W. (2018). In Silico Analysis Identifies Intestinal Transit as a Key Determinant of Systemic Bile Acid Metabolism. Front. Physiol..

[108] Berr F., Stellaard F., Pratschke E., Paumgartner G. (1989). Effects of Cholecystectomy on the Kinetics of Primary and Secondary Bile Acids. J. Clin. Investig..

[109] Barrera F., Azócar L., Molina H., Schalper K.A., Ocares M., Liberona J., Villarroel L., Pimentel F., Pérez-Ayuso R.M., Nervi F. (2015). Effect of Cholecystectomy on Bile Acid Synthesis and Circulating Levels of Fibroblast Growth Factor 19. Annu. Hepatol..

[110] Zhang Y., Liu H., Li L., Ai M., Gong Z., He Y., Dong Y., Xu S., Wang J., Jin B. (2017). Cholecystectomy Can Increase the Risk of Colorectal Cancer: A Meta-Analysis of 10 Cohort Studies. PLoS ONE.

[111] Ren X., Xu J., Zhang Y., Chen G., Zhang Y., Huang Q., Liu Y. (2020). Bacterial Alterations in Post-Cholecystectomy Patients Are Associated with Colorectal Cancer. Front. Oncol..

[112] Vinikoor L.C., Robertson D.J., Baron J.A., Silverman W.B., Sandler R.S. (2007). Cholecystectomy and the Risk of Recurrent Colorectal Adenomas. Cancer Epidemiol. Biomark. Amp. Prev..

[113] Yoshimoto S., Loo T.M., Atarashi K., Kanda H., Sato S., Oyadomari S., Iwakura Y., Oshima K., Morita H., Hattori M. (2013). Obesity-Induced Gut Microbial Metabolite Promotes Liver Cancer through Senescence Secretome. Nature.

[114] Schafer M.J., Zhang X., Kumar A., Atkinson E.J., Zhu Y., Jachim S., Mazula D.L., Brown A.K., Berning M., Aversa Z. (2020). The Senescence-Associated Secretome as an Indicator of Age and Medical Risk. JCI Insight.

[115] Devkota S., Wang Y., Musch M.W., Leone V., Fehlner-Peach H., Nadimpalli A., Antonopoulos D.A., Jabri B., Chang E.B. (2012). Dietary-Fat-Induced Taurocholic Acid Promotes Pathobiont Expansion and Colitis in Il10−/− Mice. Nature.

[116] Vacante M., Ciuni R., Basile F., Biondi A. (2020). Gut Microbiota and Colorectal Cancer Development: A Closer Look to the Adenoma-Carcinoma Sequence. Biomedicines.

[117] Baiocchi L., Zhou T., Liangpunsakul S., Lenci I., Santopaolo F., Meng F., Kennedy L., Glaser S., Francis H., Alpini G. (2019). Dual Role of Bile Acids on the Biliary Epithelium: Friend or Foe?. Int. J. Mol. Sci..

[118] Roma M.G., Sanchez Pozzi E.J. (2008). Oxidative Stress: A Radical Way to Stop Making Bile. Annu. Hepatol..

[119] Daujat-Chavanieu M., Gerbal-Chaloin S. (2020). Regulation of CAR and PXR Expression in Health and Disease. Cells.

[120] Kakiyama G., Pandak W.M., Gillevet P.M., Hylemon P.B., Heuman D.M., Daita K., Takei H., Muto A., Nittono H., Ridlon J.M. (2013). Modulation of the Fecal Bile Acid Profile by Gut Microbiota in Cirrhosis. J. Hepatol..

[121] Steib C.J., Hartmann A.C., Hesler C.V., Benesic A., Hennenberg M., Bilzer M., Gerbes A.L. (2010). Intraperitoneal LPS Amplifies Portal Hypertension in Rat Liver Fibrosis. Lab. Investig..

[122] Bräsen J.H., Mederacke Y., Schmitz J., Diahovets K., Khalifa A., Hartleben B., Person F., Wiech T., Steenbergen E., Großhennig A. (2019). Cholemic Nephropathy Causes Acute idney Injury and Is Accompanied by Loss of Aquaporin 2 in Collecting Ducts. Hepatology.

[123] Chiang J.Y.L. (2009). Bile Acids: Regulation of Synthesis. J. Lipid Res..

[124] Li T., Apte U. (2015). Bile Acid Metabolism and Signaling in Cholestasis, Inflammation, and Cancer. Advances in Pharmacology.

[125] Matsuzaki Y., Bouscarel B., Ikegami T., Honda A., Doy M., Ceryak S., Fukushima S., Yoshida S., Shoda J., Tanaka N. (2002). Selective Inhibition of CYP27A1 and of Chenodeoxycholic Acid Synthesis in Cholestatic Hamster Liver. Biochim. Biophys. Acta BBA Mol. Basis Dis..

[126] Meng L., Quezada M., Levine P., Han Y., McDaniel K., Zhou T., Lin E., Glaser S., Meng F., Francis H. (2015). Functional Role of Cellular Senescence in Biliary Injury. Am. J. Pathol..

[127] Lleo A., Leung P.S.C., Hirschfield G.M., Gershwin E.M. (2020). The Pathogenesis of Primary Biliary Cholangitis: A Comprehensive Review. Semin. Liver Dis..

[128] Pinto C., Ninfole E., Benedetti A., Maroni L., Marzioni M. (2020). Aging-Related Molecular Pathways in Chronic Cholestatic Conditions. Front. Med..

[129] Banales J.M., Huebert R.C., Karlsen T., Strazzabosco M., LaRusso N.F., Gores G.J. (2019). Cholangiocyte Pathobiology. Nat. Rev. Gastroenterol. Hepatol..

[130] Melero S., Spirlì C., Zsembery Á., Medina J.F., Joplin R.E., Duner E., Zuin M., Neuberger J.M., Prieto J., Strazzabosco M. (2002). Defective Regulation of Cholangiocyte Cl^−^/HCO^−^_3_ and Na^+^/H^+^ Exchanger Activities in Primary Biliary Cirrhosis: Defective Regulation of Cholangiocyte Cl^−^/HCO^−^_3_ and Na^+^/H^+^ Exchanger Activities in Primary Biliary Cirrhosis. Hepatology.

[131] Salas J.T., Banales J.M., Sarvide S., Recalde S., Ferrer A., Uriarte I., Oude Elferink R.P.J., Prieto J., Medina J.F. (2008). Ae2a,b-Deficient Mice Develop Antimitochondrial Antibodies and Other Features Resembling Primary Biliary Cirrhosis. Gastroenterology.

[132] Colombo C., Battezzati P., Strazzabosco M., Podda M. (1998). Liver and Biliary Problems in Cystic Fibrosis. Semin. Liver Dis..

[133] Jahan A., Chiang J.Y.L. (2005). Cytokine Regulation of Human Sterol 12α-Hydroxylase (CYP8B1) Gene. Am. J. Physiol.-Gastrointest. Liver Physiol..

[134] Dueland S., Reichen J., Everson G.T., Davis R.A. (1991). Regulation of Cholesterol and Bile Acid Homoeostasis in Bile-Obstructed Rats. Biochem. J..

[135] Song K.H., Ellis E., Strom S., Chiang J.Y.L. (2007). Hepatocyte Growth Factor Signaling Pathway Inhibits Cholesterol 7α-Hydroxylase and Bile Acid Synthesis in Human Hepatocytes. Hepatology.

[136] Lanzini A. (2003). Intestinal Absorption of the Bile Acid Analogue 75Se-Homocholic Acid-Taurine Is Increased in Primary Biliary Cirrhosis and Reverts to Normal during Ursodeoxycholic Acid Administration. Gut.

[137] Jansen P.L.M. (2018). New Therapies Target the Toxic Consequences of Cholestatic Liver Disease. Expert Rev. Gastroenterol. Hepatol..

[138] Shah R.A., Kowdley K.V. (2020). Current and Potential Treatments for Primary Biliary Cholangitis. Lancet Gastroenterol. Hepatol..

[139] Gomez-Ospina N., Potter C.J., Xiao R., Manickam K., Kim M.-S., Kim K.H., Shneider B.L., Picarsic J.L., Jacobson T.A., Zhang J. (2016). Mutations in the Nuclear Bile Acid Receptor FXR Cause Progressive Familial Intrahepatic Cholestasis. Nat. Commun..

[140] Arroyo M., Crawford J.M. (2006). Hepatitic Inherited Metabolic Disorders. Semin. Diagn. Pathol..

[141] Sangkhathat S., Laochareonsuk W., Maneechay W., Kayasut K., Chiengkriwate P. (2018). Variants Associated with Infantile Cholestatic Syndromes Detected in Extrahepatic Biliary Atresia by Whole Exome Studies: A 20-Case Series from Thailand. J. Pediatr. Genet..

[142] Massarweh N.N., El-Serag H.B. (2017). Epidemiology of Hepatocellular Carcinoma and Intrahepatic Cholangiocarcinoma. Cancer Control.

[143] Liu R., Li X., Qiang X., Luo L., Hylemon P.B., Jiang Z., Zhang L., Zhou H. (2015). Taurocholate Induces Cyclooxygenase-2 Expression via the Sphingosine 1-Phosphate Receptor 2 in a Human Cholangiocarcinoma Cell Line. J. Biol. Chem..

[144] Dai J., Wang H., Shi Y., Dong Y., Zhang Y., Wang J. (2011). Impact of Bile Acids on the Growth of Human Cholangiocarcinoma via FXR. J. Hematol. Oncol. J. Hematol. Oncol..

[145] Yang F., Huang X., Yi T., Yen Y., Moore D.D., Huang W. (2007). Spontaneous Development of Liver Tumors in the Absence of the Bile Acid Receptor Farnesoid X Receptor. Cancer Res..

[146] Chiang J.Y.L. (2015). Negative Feedback Regulation of Bile Acid Metabolism: Impact on Liver Metabolism and Diseases: Hepatology elsewhere. Hepatology.

[147] Cave M.C., Clair H.B., Hardesty J.E., Falkner K.C., Feng W., Clark B.J., Sidey J., Shi H., Aqel B.A., McClain C.J. (2016). Nuclear Receptors and Nonalcoholic Fatty Liver Disease. Biochim. Biophys. Acta BBA Gene Regul. Mech..

[148] Urso A., D’Ovidio F., Xu D., Emala C.W., Bunnett N.W., Perez-Zoghbi J.F. (2020). Bile Acids Inhibit Cholinergic Constriction in Proximal and Peripheral Airways from Humans and Rodents. Am. J. Physiol. Lung Cell. Mol. Physiol..

[149] Urso A., Perez-Zoghbi J., Nandakumar R., Cremers S., Bunnett N., Emala C., D’Ovidio F. (2019). Aspirated Bile Acids Affect Lung Immunity and Function. Transplantation.

[150] Chen B., You W.J., Liu X.Q., Xue S., Qin H., Jiang H.D. (2017). Chronic Microaspiration of Bile Acids Induces Lung Fibrosis through Multiple Mechanisms in Rats. Clin. Sci..

[151] Kjærgaard K., Frisch K., Sørensen M., Munk O.L., Hofmann A.F., Horsager J., Schacht A.C., Erickson M., Shapiro D., Keiding S. (2021). Obeticholic Acid Improves Hepatic Bile Acid Excretion in Patients with Primary Biliary Cholangitis. J. Hepatol..

[152] Hofmann A.F. (1999). The Continuing Importance of Bile Acids in Liver and Intestinal Disease. Arch. Intern. Med..

[153] Khurana S., Raina H., Pappas V., Raufman J.-P., Pallone T.L. (2012). Effects of Deoxycholylglycine, a Conjugated Secondary Bile Acid, on Myogenic Tone and Agonist-Induced Contraction in Rat Resistance Arteries. PLoS ONE.

[154] Nakajima T., Okuda Y., Chisaki K., Shin W.-S., Iwasawa K., Morita T., Matsumoto A., Suzuki J., Suzuki S., Yamada N. (2000). Bile Acids Increase Intracellular Ca^2+^ Concentration and Nitric Oxide Production in Vascular Endothelial Cells: Bile Acids and Endothelial Cells. Br. J. Pharmacol..

[155] Alon U., Berant M., Mordechovitz D., Hashmonai M., Better O.S. (1982). Effect of Isolated Cholaemia on Systemic Haemodynamics and Kidney Function in Conscious Dogs. Clin. Sci. Lond. Engl..

[156] Bomzon A., Finberg J.P., Tovbin D., Naidu S.G., Better O.S. (1984). Bile Salts, Hypotension and Obstructive Jaundice. Clin. Sci. Lond. Engl..

[157] Pak J.M., Lee S.S. (1993). Vasoactive Effects of Bile Salts in Cirrhotic Rats: In Vivo and in Vitro Studies. Hepatololy.

[158] He F., Li J., Mu Y., Kuruba R., Ma Z., Wilson A., Alber S., Jiang Y., Stevens T., Watkins S. (2006). Downregulation of Endothelin-1 by Farnesoid X Receptor in Vascular Endothelial Cells. Circ. Res..

[159] Li J., Wilson A., Kuruba R., Zhang Q., Gao X., He F., Zhang L.M., Pitt B.R., Xie W., Li S. (2008). FXR-Mediated Regulation of ENOS Expression in Vascular Endothelial Cells. Cardiovasc. Res..

[160] Zhang Q., He F., Kuruba R., Gao X., Wilson A., Li J., Billiar T.R., Pitt B.R., Xie W., Li S. (2008). FXR-mediated regulation of angiotensin type 2 receptor expression in vascular smooth muscle cells. Cardiovasc. Res..

[161] Zhang R., Ma W.Q., Fu M.J., Li J., Hu C.H., Chen Y., Zhou M.M., Gao Z.J., He Y.L. (2021). Overview of Bile Acid Signaling in the Cardiovascular System. World J. Clin. Cases.

[162] Pols T.W.H. (2014). TGR5 in Inflammation and Cardiovascular Disease. Biochem. Soc. Trans..

[163] Charach G., Rabinovich A., Argov O., Weintraub M., Rabinovich P. (2012). The Role of Bile Acid Excretion in Atherosclerotic Coronary Artery Disease. Int. J. Vasc. Med..

[164] Li W., Shu S., Cheng L., Hao X., Wang L., Wu Y., Yuan Z., Zhou J. (2020). Fasting Serum Total Bile Acid Level Is Associated with Coronary Artery Disease, Myocardial Infarction and Severity of Coronary Lesions. Atherosclerosis.

[165] Hanniman E.A., Lambert G., McCarthy T.C., Sinal C.J. (2005). Loss of Functional Farnesoid X Receptor Increases Atherosclerotic Lesions in Apolipoprotein E-Deficient Mice. J. Lipid Res..

[166] Ma Z., Lee S.S., Meddings J.B. (1997). Effects of Altered Cardiac Membrane Fluidity on β-Adrenergic Receptor Signalling in Rats with Cirrhotic Cardiomyopathy. J. Hepatol..

[167] Joubert P. (1978). An in vivo investigation of the negative chronotropic effect of cholic acid in the rat. Clin. Exp. Pharmacol. Physiol..

[168] Raufman J.P., Chen Y., Zimniak P., Cheng K. (2002). Deoxycholic Acid Conjugates Are Muscarinic Cholinergic Receptor Antagonists. Pharmacology.

[169] Gao J., Yuan G., Xu Z., Lan L., Xin W. (2021). Chenodeoxycholic and Deoxycholic Acids Induced Positive Inotropic and Negative Chronotropic Effects on Rat Heart. Naunyn. Schmiedebergs Arch. Pharmacol..

[170] Schultz F., Hasan A., Alvarez-Laviada A., Miragoli M., Bhogal N., Wells S., Poulet C., Chambers J., Williamson C., Gorelik J. (2016). The Protective Effect of Ursodeoxycholic Acid in an in Vitro Model of the Human Fetal Heart Occurs via Targeting Cardiac Fibroblasts. Prog. Biophys. Mol. Biol..

[171] von Haehling S., Schefold J.C., Jankowska E.A., Springer J., Vazir A., Kalra P.R., Sandek A., Fauler G., Stojakovic T., Trauner M. (2012). Ursodeoxycholic Acid in Patients with Chronic Heart Failure: A Double-Blind, Randomized, Placebo-Controlled, Crossover Trial. J. Am. Coll. Cardiol..

[172] Mayerhofer C.C.K., Ueland T., Broch K., Vincent R.P., Cross G.F., Dahl C.P., Aukrust P., Gullestad L., Hov J.R., Trøseid M. (2017). Increased Secondary/Primary Bile Acid Ratio in Chronic Heart Failure. J. Card. Fail..

[173] Gorelik J., Shevchuk A., de Swiet M., Lab M., Korchev Y., Williamson C. (2004). Comparison of the Arrhythmogenic Effects of Tauro- and Glycoconjugates of Cholic Acid in an in Vitro Study of Rat Cardiomyocytes. BJOG Int. J. Obstet. Gynaecol..

[174] Desai M.S., Shabier Z., Taylor M., Lam F., Thevananther S., Kosters A., Karpen S.J. (2010). Hypertrophic Cardiomyopathy and Dysregulation of Cardiac Energetics in a Mouse Model of Biliary Fibrosis. Hepatology.

[175] Kitai T., Tang W.H.W. (2018). Gut Microbiota in Cardiovascular Disease and Heart Failure. Clin. Sci..

[176] Inagaki T., Moschetta A., Lee Y.K., Peng L., Zhao G., Downes M., Yu R.T., Shelton J.M., Richardson J.A., Repa J.J. (2006). Regulation of Antibacterial Defense in the Small Intestine by the Nuclear Bile Acid Receptor. Proc. Natl. Acad. Sci. USA.

[177] Hofmann A.F., Eckmann L. (2006). How Bile Acids Confer Gut Mucosal Protection against Bacteria. Proc. Natl. Acad. Sci. USA.

[178] Islam K.B.M.S., Fukiya S., Hagio M., Fujii N., Ishizuka S., Ooka T., Ogura Y., Hayashi T., Yokota A. (2011). Bile Acid Is a Host Factor That Regulates the Composition of the Cecal Microbiota in Rats. Gastroenterology.

[179] Ridlon J.M., Kang D.J., Hylemon P.B. (2010). Isolation and Characterization of a Bile Acid Inducible 7α-Dehydroxylating Operon in Clostridium Hylemonae TN271. Anaerobe.

[180] Browne H.P., Forster S.C., Anonye B.O., Kumar N., Neville B.A., Stares M.D., Goulding D., Lawley T.D. (2016). Culturing of ‘Unculturable’ Human Microbiota Reveals Novel Taxa and Extensive Sporulation. Nature.

[181] Philipp B. (2011). Bacterial Degradation of Bile Salts. Appl. Microbiol. Biotechnol..

[182] Zanetti M. (2004). Cathelicidins, Multifunctional Peptides of the Innate Immunity. J. Leukoc. Biol..

[183] Joyce S.A., MacSharry J., Casey P.G., Kinsella M., Murphy E.F., Shanahan F., Hill C., Gahan C.G.M. (2014). Regulation of Host Weight Gain and Lipid Metabolism by Bacterial Bile Acid Modification in the Gut. Proc. Natl. Acad. Sci. USA.

[184] Pols T.W.H., Noriega L.G., Nomura M., Auwerx J., Schoonjans K. (2011). The Bile Acid Membrane Receptor TGR5 as an Emerging Target in Metabolism and Inflammation. J. Hepatol..

[185] de Aguiar Vallim T.Q., Tarling E.J., Edwards P.A. (2013). Pleiotropic Roles of Bile Acids in Metabolism. Cell Metab..

[186] Porez G., Prawitt J., Gross B., Staels B. (2012). Bile Acid Receptors as Targets for the Treatment of Dyslipidemia and Cardiovascular Disease. J. Lipid Res..

[187] Yamaoka-Tojo M., Tojo T., Izumi T. (2008). Beyond Cholesterol Lowering: Pleiotropic Effects of Bile Acid Binding Resins Against Cardiovascular Disease Risk Factors in Patients with Metabolic Syndrome. Curr. Vasc. Pharmacol..

[188] Ali A.H., Carey E.J., Lindor K.D. (2015). Recent Advances in the Development of Farnesoid X Receptor Agonists. Ann. Transl. Med..

[189] Watanabe M., Houten S.M., Wang L., Moschetta A., Mangelsdorf D.J., Heyman R.A., Moore D.D., Auwerx J. (2004). Bile Acids Lower Triglyceride Levels via a Pathway Involving FXR, SHP, and SREBP-1c. J. Clin. Investig..

[190] Chiang J.Y.L. (2013). Bile Acid Metabolism and Signaling. Compr. Physiol..

[191] Smith Z., Ryerson D., Kemper J.K. (2013). Epigenomic Regulation of Bile Acid Metabolism: Emerging Role of Transcriptional Cofactors. Mol. Cell. Endocrinol..

[192] Jia X., Suzuki Y., Naito H., Yetti H., Kitamori K., Hayashi Y., Kaneko R., Nomura M., Yamori Y., Zaitsu K. (2014). A Possible Role of Chenodeoxycholic Acid and Glycine-Conjugated Bile Acids in Fibrotic Steatohepatitis in a Dietary Rat Model. Dig. Dis. Sci..

[193] Quintero P., Pizarro M., Solís N., Arab J.P., Padilla O., Riquelme A., Arrese M. (2014). Bile Acid Supplementation Improves Established Liver Steatosis in Obese Mice Independently of Glucagon-like Peptide-1 Secretion. J. Physiol. Biochem..

[194] Woolbright B.L. (2012). Novel Insight into Mechanisms of Cholestatic Liver Injury. World J. Gastroenterol..

[195] Kirbas O., Biberoglu E.H., Kirbas A., Daglar K., Kurmus O., Danisman N., Biberoglu K. (2015). Evaluation of Ventricular Repolarization in Pregnant Women with Intrahepatic Cholestasis. Int. J. Cardiol..

[196] Paumgartner G. (2002). Ursodeoxycholic Acid in Cholestatic Liver Disease: Mechanisms of Action and Therapeutic Use Revisited. Hepatology.

[197] Häussinger D., Kordes C. (2017). Mechanisms of Tauroursodeoxycholate-Mediated Hepatoprotection. Dig. Dis..

[198] Nevens F., Andreone P., Mazzella G., Strasser S.I., Bowlus C., Invernizzi P., Drenth J.P.H., Pockros P.J., Regula J., Beuers U. (2016). A Placebo-Controlled Trial of Obeticholic Acid in Primary Biliary Cholangitis. N. Engl. J. Med..

[199] Hirschfield G.M., Mason A., Luketic V., Lindor K., Gordon S.C., Mayo M., Kowdley K.V., Vincent C., Bodhenheimer H.C., Parés A. (2015). Efficacy of Obeticholic Acid in Patients with Primary Biliary Cirrhosis and Inadequate Response to Ursodeoxycholic Acid. Gastroenterology.

[200] Úbeda M., Lario M., Muñoz L., Borrero M.-J., Rodríguez-Serrano M., Sánchez-Díaz A.-M., Del Campo R., Lledó L., Pastor Ó., García-Bermejo L. (2016). Obeticholic Acid Reduces Bacterial Translocation and Inhibits Intestinal Inflammation in Cirrhotic Rats. J. Hepatol..

[201] Laleman W., Trebicka J., Verbeke L. (2016). Evolving Insights in the Pathophysiology of Complications of Cirrhosis: The Farnesoid X Receptor (FXR) to the Rescue?. Hepatology.

[202] Vignozzi L., Morelli A., Cellai I., Filippi S., Comeglio P., Sarchielli E., Maneschi E., Vannelli G.B., Adorini L., Maggi M. (2017). Cardiopulmonary Protective Effects of the Selective FXR Agonist Obeticholic Acid in the Rat Model of Monocrotaline-Induced Pulmonary Hypertension. J. Steroid Biochem. Mol. Biol..

[203] Voiosu A., Wiese S., Voiosu T., Bendtsen F., Møller S. (2017). Bile Acids and Cardiovascular Function in Cirrhosis. Liver Int..

[204] Staley C., Weingarden A.R., Khoruts A., Sadowsky M.J. (2017). Interaction of Gut Microbiota with Bile Acid Metabolism and Its Influence on Disease States. Appl. Microbiol. Biotechnol..

[205] Li G., Kong B., Zhu Y., Zhan L., Williams J.A., Tawfik O., Kassel K.M., Luyendyk J.P., Wang L., Guo G.L. (2013). Small Heterodimer Partner Overexpression Partially Protects against Liver Tumor Development in Farnesoid X Receptor Knockout Mice. Toxicol. Appl. Pharmacol..

[206] Jia W., Xie G., Jia W. (2018). Bile Acid–Microbiota Crosstalk in Gastrointestinal Inflammation and Carcinogenesis. Nat. Rev. Gastroenterol. Hepatol..

[207] Farhana L., Nangia-Makker P., Arbit E., Shango K., Sarkar S., Mahmud H., Hadden T., Yu Y., Majumdar A.P.N. (2016). Bile Acid: A Potential Inducer of Colon Cancer Stem Cells. Stem Cell Res. Ther..

[208] Ridlon J.M., Wolf P.G., Gaskins H.R. (2016). Taurocholic Acid Metabolism by Gut Microbes and Colon Cancer. Gut Microbes.

[209] Hashimoto N. (2012). Expression of COX2 and P53 in Rat Esophageal Cancer Induced by Reflux of Duodenal Contents. ISRN Gastroenterol..

[210] Hashimoto N. (2018). Effect of Pancreatic Juice and Bile Reflux to the Development of Esophageal Carcinogenesis in Rat Model. Clin. Oncol..

[211] Weingarden A.R., Dosa P.I., DeWinter E., Steer C.J., Shaughnessy M.K., Johnson J.R., Khoruts A., Sadowsky M.J. (2016). Changes in Colonic Bile Acid Composition Following Fecal Microbiota Transplantation Are Sufficient to Control Clostridium Difficile Germination and Growth. PLoS ONE.

[212] Weingarden A.R., Chen C., Zhang N., Graiziger C.T., Dosa P.I., Steer C.J., Shaughnessy M.K., Johnson J.R., Sadowsky M.J., Khoruts A. (2016). Ursodeoxycholic Acid Inhibits Clostridium Difficile Spore Germination and Vegetative Growth and Prevents the Recurrence of Ileal Pouchitis Associated with the Infection. J. Clin. Gastroenterol..

[213] Huo X. (2015). Therapeutic and Chemopreventive Effects of Ursodeoxycholic Acid (UDCA): Potential Role in Patients with Barrett’s Esophagus. Gastro Open J..

[214] Woods D.F., Flynn S., Caparrós-Martín J.A., Stick S.M., Reen F.J., O’Gara F. (2021). Systems Biology and Bile Acid Signalling in Microbiome-Host Interactions in the Cystic Fibrosis Lung. Antibiotics.

[215] McMillin M., DeMorrow S. (2016). Effects of Bile Acids on Neurological Function and Disease. FASEB J. Off. Publ. Fed. Am. Soc. Exp. Biol..

[216] Bajaj J.S., Ridlon J.M., Hylemon P.B., Thacker L.R., Heuman D.M., Smith S., Sikaroodi M., Gillevet P.M. (2012). Linkage of Gut Microbiome with Cognition in Hepatic Encephalopathy. Am. J. Physiol. Gastrointest. Liver Physiol..

[217] Bajaj J.S., Heuman D.M., Hylemon P.B., Sanyal A.J., White M.B., Monteith P., Noble N.A., Unser A.B., Daita K., Fisher A.R. (2014). Altered Profile of Human Gut Microbiome Is Associated with Cirrhosis and Its Complications. J. Hepatol..

[218] Attili A.F., Angelico M., Cantafora A., Alvaro D., Capocaccia L. (1986). Bile Acid-Induced Liver Toxicity: Relation to the Hydrophobic-Hydrophilic Balance of Bile Acids. Med. Hypotheses.

[219] Zimber A., Zusman I., Bentor R., Pinus H. (1991). Effects of Lithocholic Acid Exposure throughout Pregnancy on Late Prenatal and Early Postnatal Development in Rats. Teratology.

[220] Debruyne P.R., Bruyneel E.A., Li X., Zimber A., Gespach C., Mareel M.M. (2001). The Role of Bile Acids in Carcinogenesis. Mutat. Res..

[221] Costarelli V., Sanders T.a.B. (2002). Plasma Deoxycholic Acid Concentration Is Elevated in Postmenopausal Women with Newly Diagnosed Breast Cancer. Eur. J. Clin. Nutr..

[222] Zdebska E., Iolascon A., Spychalska J., Perrotta S., Lanzara C., Smolenska-Sym G., Koscielak J. (2007). Abnormalities of erythrocyte glycoconjugates are identical in two families with congenital dyserythropoietic anemia type II with different chromosomal localizations of the disease gene. Haematologica.

[223] Monte M.J., Marin J.J.G., Antelo A., Vazquez-Tato J. (2009). Bile Acids: Chemistry, Physiology, and Pathophysiology. World J. Gastroenterol..

